# Supplementary material for: Donor leukocyte telomere length emerges as a prognostic factor for transplantation outcomes
Source: Innovation (Camb). 2025 Oct 24;7(3):101147. doi: 10.1016/j.xinn.2025.101147 (PMC12957556; doi:10.1016/j.xinn.2025.101147)
Supplement: Document S1. Tables S1–S3 [file mmc1.pdf]

The Innovation, Volume 7

Supplemental Information

Donor leukocyte telomere length emerges as a prognostic factor for transplantation outcomes

Hengwei Wu, Jing Yu, Yang Cao, Ruowen Wei, Wei Shi, Yunxian Yu, Zhuoyue Shi, Jimin Shi, Yi Luo, Jian Yu, Xiaoyu Lai, Lizhen Liu, Yamin Tan, Huarui Fu, Pengxu Qian, Wenming Shi, Zhongzheng Zheng, Xiaoxia Hu, He Huang, and Yanmin Zhao

Document S1

Definitions

CR, complete remission

CR is defined as the presence of fewer than 5% leukemic blasts in the bone marrow, with no evidence of leukemic cells in the peripheral blood or at extramedullary sites.

R-DRI, refined Disease Risk Index

R-DRI is a clinically validated stratification system used to predict prognosis in patients undergoing allogeneic hematopoietic stem cell transplantation, particularly those with hematologic malignancies. R-DRI categorizes patients into four risk groups—low, intermediate, high, and very high—based on disease type, disease status, and prognostic factors such as cytogenetic and molecular abnormalities.[S1]

BMI, body mass index

BMI was calculated as weight in kilograms divided by the square of height in meters (kg/m²). Weight and height were measured by trained healthcare professionals following standardized protocols established by the World Health Organization. Participants were measured without shoes and in light clothing. Weight was recorded to the nearest 0.1 kg using a calibrated beam scale, and height to the nearest 0.2 cm using a portable stadiometer. Each measurement was taken three times, and the average value was used in the calculation. Based on BMI, participants were classified as underweight (< 18.5 kg/m²), normal weight (18.5–23.9 kg/m²), overweight (24.0–27.9 kg/m²), or obese (≥ 28.0 kg/m²).[S2, S3]

Reference

[S1]. Armand P, Kim HT, Logan BR, et al. Validation and refinement of the Disease Risk Index for allogeneic stem cell transplantation. *Blood*. 2014 Jun 5;123(23):3664-71.

[S2]. WHO Expert Consultation. Appropriate body-mass index for Asian populations and its implications for policy and intervention strategies. *Lancet*. 2004;363:157–163.

[S3]. Zhou BF; Cooperative Meta-Analysis Group of the Working Group on Obesity in China. Predictive values of body mass index and waist circumference for risk factors of certain related diseases in Chinese adults--study on optimal cut-off points of body mass index and waist circumference in Chinese adults. *Biomed Environ Sci*. 2002 Mar;15(1):83-96.

Supplemental Tables

Table S1. Transplantation plan

**Transplantation plan**	
Conditioning regimen	
Myeloablative regimen	ARAC2Bu3Cy2 / Bu4Cy2 / Flu5Bu4 / Flu5Bu2Mel100
Reduced intensity regimen	Flu6Bu3 / Flu6Bu2 / Flu6Mel140–120
T cell depletion regimen (in vivo)	
Haploidentical HSCT	Anti–T lymphocyte globulin (Grafalon®, 10–15 mg/kg, intravenous infusion, days –5 to –2) or anti-thymocyte globulin (Thymoglobulin®, 6–7.5 mg/kg, intravenous infusion, days –5 to –2)
Matched related HSCT	Thymoglobulin® (4.5 mg/kg, intravenous infusion, days –5 to –2) for patients or donors aged > 40 years
Unrelated HSCT	Thymoglobulin® (4.5–7.5 mg/kg, intravenous infusion, days –5 or –4 to –2)
Peripheral blood stem cell mobilization regimen	
All donors	Granulocyte colony-stimulating factor, 7.5–10 μg/kg/day, subcutaneous injection for 4–5 consecutive days, followed by peripheral blood stem cell collection

ARAC, Cytarabine; Bu, Busulfan; Cy, Cyclophosphamide; Flu, Fludarabine; HSCT, hematopoietic stem cell transplantation; Mel, Melphalan.

Table S2. Baseline patient and transplant characteristics by donor leukocyte telomere length quartiles (Q1–Q4) in the discovery cohort

Donor LTL, quartiles	Q1	Q2	Q3	Q4	*p* value
n	269 (26%)	258 (25%)	262 (25%)	260 (25%)	
Donor age at transplantation, yr	40 (29–47)	35 (25–45)	34(25–44)	30 (23–41)	< 0.001
Donor sex					0.002
Female	74 (28%)	91 (35%)	97 (37%)	120 (46%)	
Male	195 (72%)	167 (65%)	165 (63%)	140 (54%)	
Patient age at transplantation, yr	33 (24–46)	34 (25–46)	35 (26–50)	38 (29–49)	0.018
Patient age at transplantation, yr					0.09
< 18	30 (11%)	20 (8%)	25 (10%)	12 (5%)	
≥ 18	239 (89%)	238 (92%)	237 (90%)	248 (95%)	
Patient sex					0.80
Female	123 (46%)	115 (45%)	127 (48%)	124 (48%)	
Male	146 (54%)	143 (55%)	135 (52%)	136 (52%)	
From diagnosis to transplantation, mo	7 (5–10)	7 (5–10)	7 (5–10)	6 (5–10)	0.016
Diagnosis					0.15
Acute myeloid leukemia	138 (51%)	131(51%)	137 (52%)	159 (61%)	
Acute lymphocytic leukemia	124 (46%)	123 (48%)	119 (45%)	100 (39%)	
Mixed phenotype acute leukemia	7 (3%)	4 (2%)	6 (2%)	1 (0%)	
Refined Disease Risk Index					0.09
Low and intermediate risk	187 (70%)	187 (72%)	189 (72%)	162 (62%)	
High and very high risk	82 (30%)	71 (28%)	73 (28%)	98 (38%)	
Remission status					0.013
CR, MRD negative	200 (74%)	192 (74%)	213 (81%)	221 (85%)	
CR, MRD positive	43 (16%)	49 (19%)	32 (12%)	18 (7%)	
Active disease	26 (10%)	17 (7%)	17 (7%)	21 (8%)	

Patient BMI at transplantation					0.80
Underweight/normal	187 (70%)	176 (68%)	170 (65%)	179 (69%)	
Overweight/obese	82 (30%)	82 (32%)	92 (35%)	81 (31%)	
Donor type					0.80
Matched sibling	35 (13%)	34 (13%)	36 (14%)	42 (16%)	
Haploidentical donor	212 (79%)	195 (76%)	201 (77%)	191 (74%)	
Matched unrelated donor	22 (8%)	29 (11%)	25 (10%)	27 (10%)	
Conditioning regimen					0.75
Myeloablative	250 (93%)	242 (94%)	246 (94%)	235 (90%)	
Reduced intensity	19 (7%)	16 (6%)	16 (6%)	25 (10%)	
Donor-recipient sex					0.020
Female to female	38 (14%)	41 (16%)	48 (18%)	54 (21%)	
Female to male	36 (13%)	50 (19%)	49 (19%)	66 (25%)	
Male to male	110 (41%)	93 (36%)	86 (33%)	70 (27%)	
Male to female	85 (32%)	74 (29%)	79 (30%)	70 (27%)	
ABO blood type match					0.76
Matched	143 (53%)	129 (50%)	137 (52%)	129 (50%)	
Minor mismatched	54 (20%)	62 (24%)	56 (21%)	59 (23%)	
Major mismatched	52 (19%)	56 (22%)	52 (20%)	50 (19%)	
Bidirectional mismatch	20 (7%)	11 (4%)	17 (7%)	22 (8%)	
Mononuclear cells, $\times 10^8$/kg	12.5 (9.5–18.3)	12.6 (8.5–17.3)	12.3 (8.5–17.7)	10.1 (6.8–14.7)	< 0.001
$CD34^+$ cells, $\times 10^6$/kg	5.2 (3.5–7.3)	5.3 (3.9–7.6)	5.1 (3.4–6.8)	4.5 (3.1–6.6)	0.018
Follow-up, yr	2.1 (1.3–3.7)	2.3 (1.3–3.6)	2.1 (1.3–3.2)	1.6 (1.2–2.9)	< 0.001
Relapse	71 (26%)	47 (18%)	48 (18%)	49(19%)	0.05
Death	81 (30%)	53 (21%)	55 (21%)	51 (20%)	0.025

NOTE. Data are n (%) or median (IQR[interquartile range]). *p* values were false discovery rate-adjusted (Benjamini–Hochberg correction).

BMI, body mass index; CR, complete remission; LTL, leukocyte telomere length; MRD, measurable residual disease.

Table S3. Patient and donor characteristics in the discovery vs. replication cohort

	Discovery cohort N = 1049	Replication cohort N = 153	*p* value
Donor age at transplantation, yr	34 (25–45)	43 (33–50)	< 0.001
Donor sex			0.06
Female	382 (36%)	44 (29%)	
Male	667 (64%)	109 (71%)	
Patient age at transplantation, yr	35 (26–48)	34 (23–47)	0.16
Patient sex			0.27
Female	489 (47%)	64 (42%)	
Male	560 (53%)	89 (58%)	
From diagnosis to transplantation, mo	7 (5–10)	6 (4–8)	< 0.001
Diagnosis			0.011
Acute myeloid leukemia	565 (54%)	94 (61%)	
Acute lymphocytic leukemia	466 (44%)	52 (34%)	
Mixed phenotype acute leukemia	18 (2%)	7 (5%)	
Refined Disease Risk Index			0.54
Low and intermediate risk	725 (69%)	102 (67%)	
High and very high risk	324 (31%)	51 (33%)	
Remission status			0.010
CR, MRD negative	826 (79%)	104 (68%)	
CR, MRD positive	142 (13%)	29 (19%)	
Active disease	81 (8%)	20 (13%)	
Patient BMI at transplantation			0.77
Underweight/normal	712 (68%)	102 (67%)	
Overweight/obese	337 (32%)	51 (33%)	
Donor type			0.16
Matched sibling	147 (14%)	20 (13%)	
Haploidentical donor	799 (76%)	125 (82%)	
Matched unrelated donor	103 (10%)	8 (5%)	
Conditioning regimen			0.05
Myeloablative	973 (93%)	135 (88%)	
Reduced intensity	76 (7%)	18 (12%)	
Donor-recipient sex			0.047
Female to female	181 (17%)	18 (12%)	
Female to male	201 (19%)	37 (24%)	
Male to male	359 (34%)	63 (41%)	
Male to female	308 (30%)	35 (23%)	
ABO blood type match			0. 55
Matched	538 (51%)	83 (54%)	
Minor mismatched	231 (22%)	26 (17%)	
Major mismatched	210 (20%)	34 (22%)	
Bidirectional mismatch	70 (7%)	10 (7%)	
Mononuclear cells, × 10^8/kg	12.0 (8.0–17.0)	14.0 (11.0–18.1)	< 0.001

CD34$^+$ cells, × 10^6/kg	5.0 (3.1–7.0)	7.0 (5.0–9.0)	< 0.001
Follow-up, yr	2.0 (1.3–3.4)	2.3 (1.3–2.6)	0.06
Relapse	215 (21%)	28 (18%)	0.53
Death	240 (23%)	28 (18%)	0.20

Data are n (%) or median (IQR[interquartile range]).
BMI, body mass index; CR, complete remission; MRD, measurable residual disease.
