# Supplementary material for: Donor leukocyte telomere length emerges as a prognostic factor for transplantation outcomes
Source: Innovation (Camb). 2025 Oct 24;7(3):101147. doi: 10.1016/j.xinn.2025.101147 (PMC12957556; doi:10.1016/j.xinn.2025.101147)

Document S2

Supplemental Figures

Figure S1. Diagram of study design

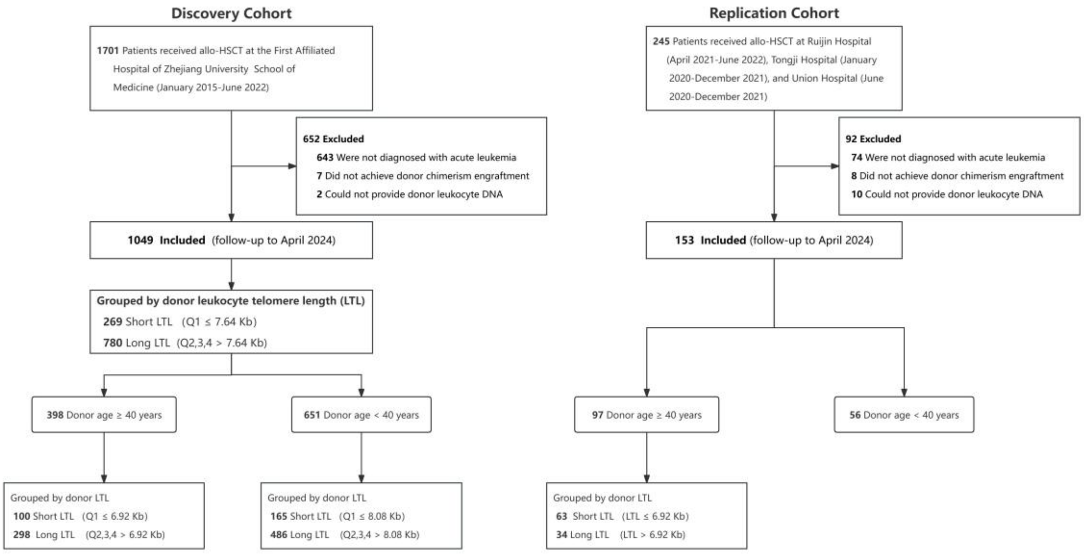

Figure S2. Association between donor leukocyte telomere length and mortality risk

(A) The restricted cubic spline plot illustrates the relationship between donor leukocyte telomere length and recipient mortality risk.
(B) Overall survival by donor leukocyte telomere length quartiles (Q1, Q2, Q3, and Q4).

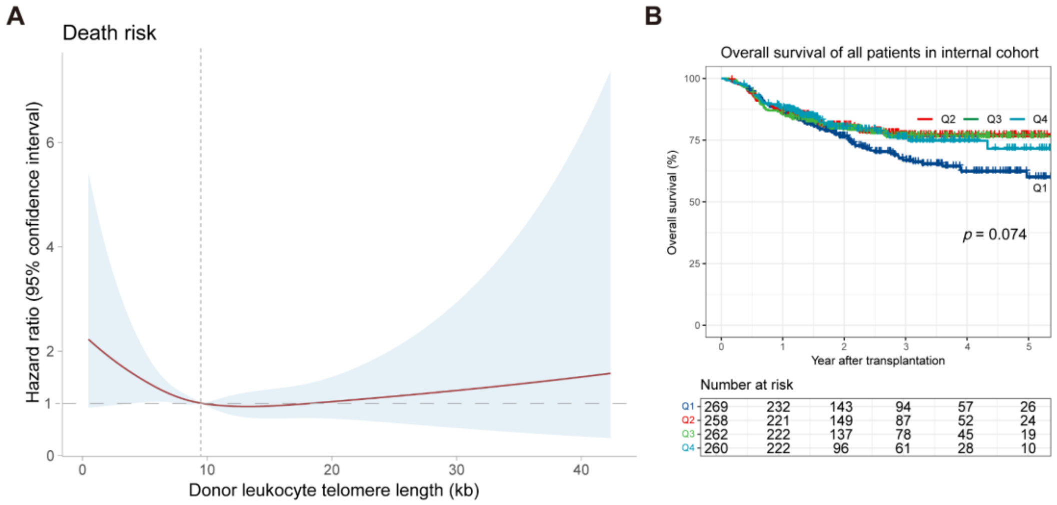

Figure S3. Secondary endpoints of all discovery cohort recipients by donor leukocyte telomere length

Cumulative incidence of (A) neutrophil engraftment, (B) platelet engraftment, (C) overall acute GVHD, (D) grade II–IV acute GVHD, (E) grade III–IV acute GVHD, (F) overall chronic GVHD, (G) moderate to severe chronic GVHD, and (H) non-relapse mortality. Probabilities of (I) GVHD-free and relapse-free survival.

GVHD, graft-versus-host disease; LTL, leukocyte telomere length.

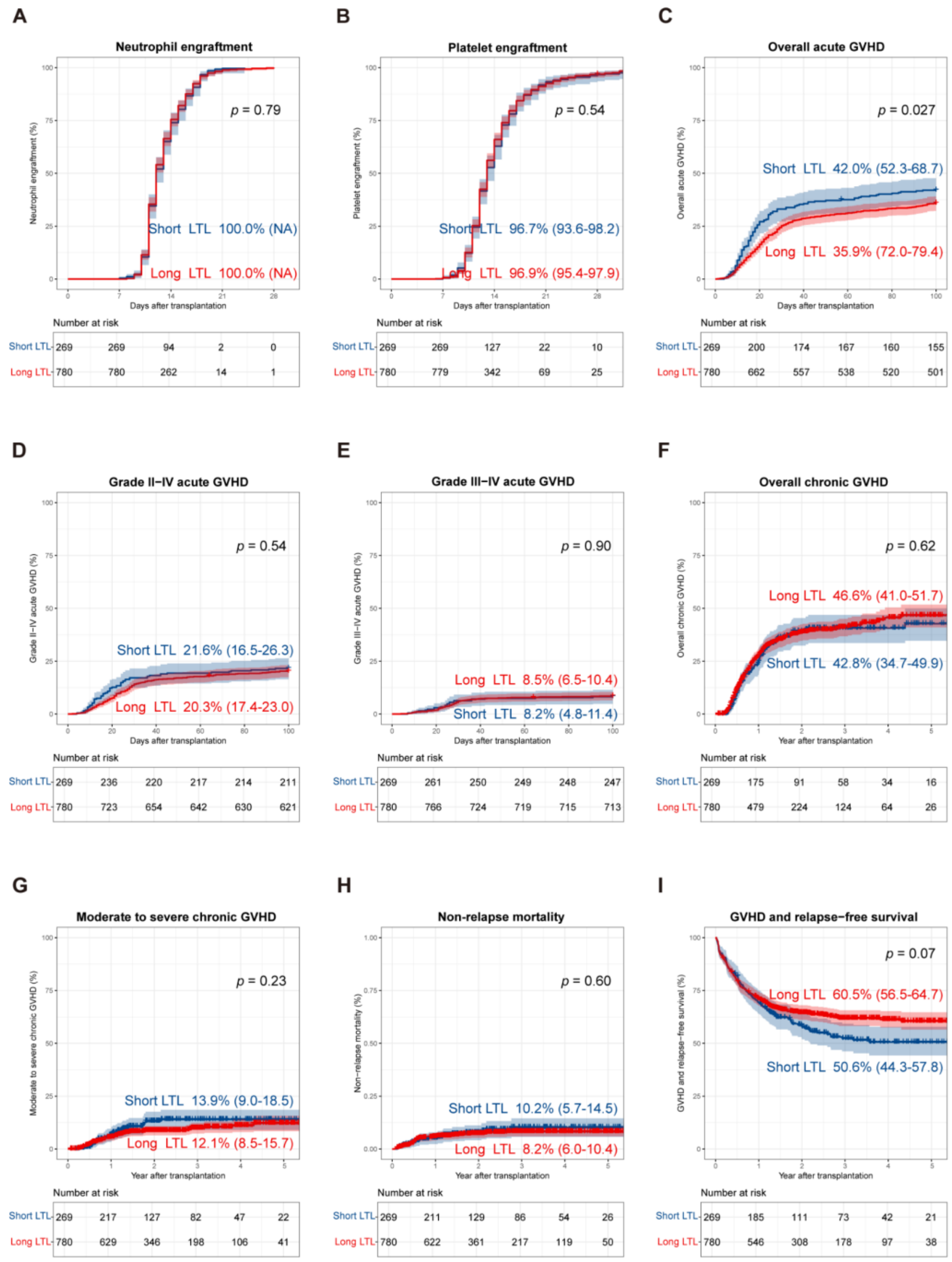

Figure S4. Distribution of donor leukocyte telomere length to telomere-related gene mutations among donors aged ≥ 40 in the discovery cohort

The blue dashed line represents the median leukocyte telomere length of mutant individuals, with genes arranged from shortest to longest based on this median. The grey area indicates the short leukocyte telomere length (Q1), while the blue area represents the long leukocyte telomere length (Q2, Q3, Q4).

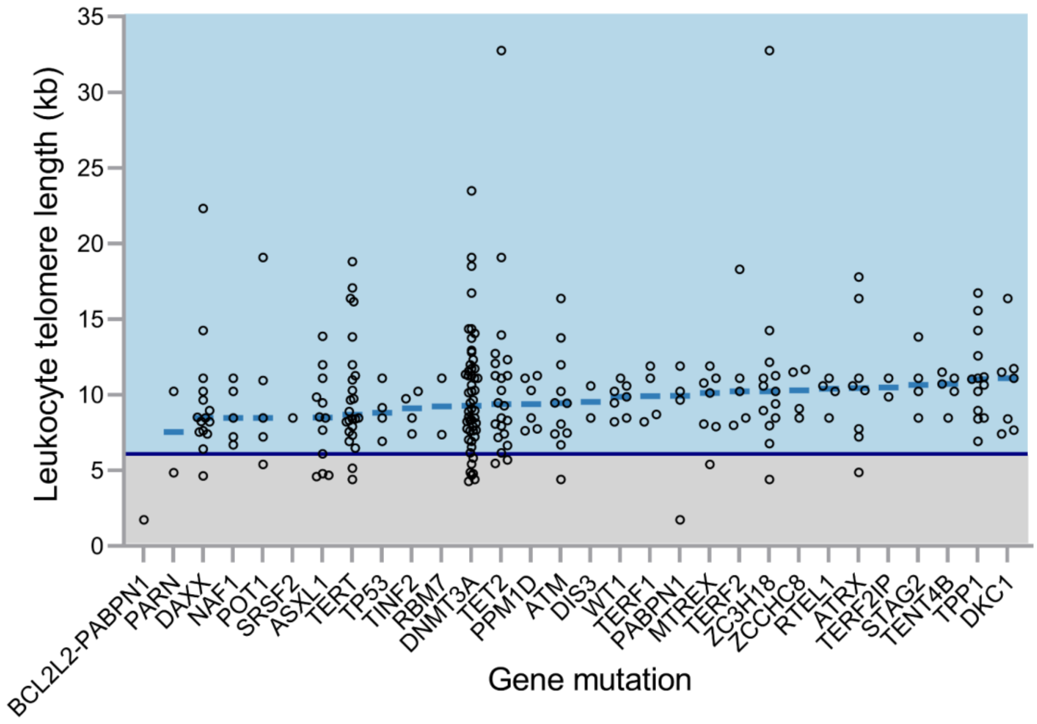

Figure S5. Mass cytometry immuno-cell profiling

(A) Abundance of different clusters across all donor groups.

(B) Cluster percentages across groups.

(C) Marker profiles for each cluster.

(D) T-SNE plots showing the distribution of clusters across groups.

(E) Differences in cluster abundance between groups.

LTL, leukocyte telomere length.

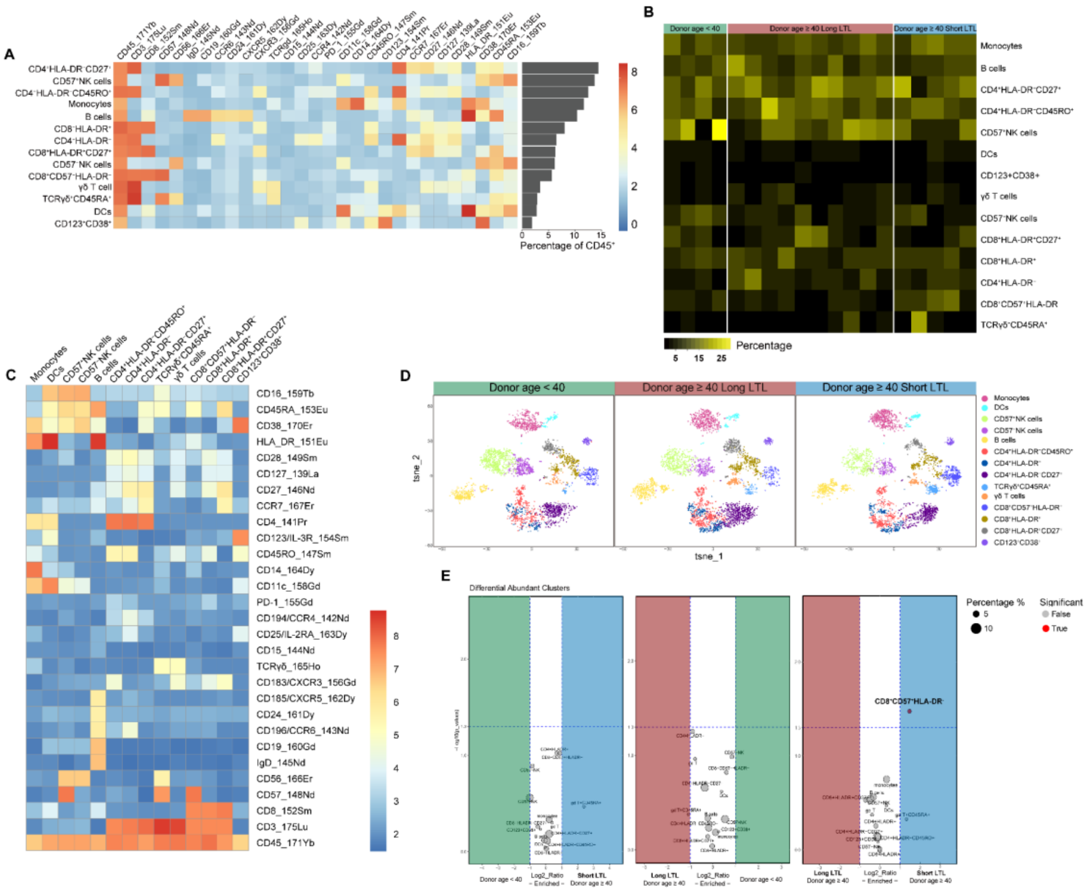

Figure S6. Multivariable models for the primary endpoints of recipients in the replication cohort

(A) Fine–Gray proportional hazard model for analyzing the cumulative incidence of relapse.

Multivariable Cox regression models for (B) overall survival and (C) relapse-free survival.

aHR, adjusted hazard ratio; CI, confidence interval; CR, complete remission; GVHD, graft-versus-host disease; LTL, leukocyte telomere length; MRD, measurable residual disease.

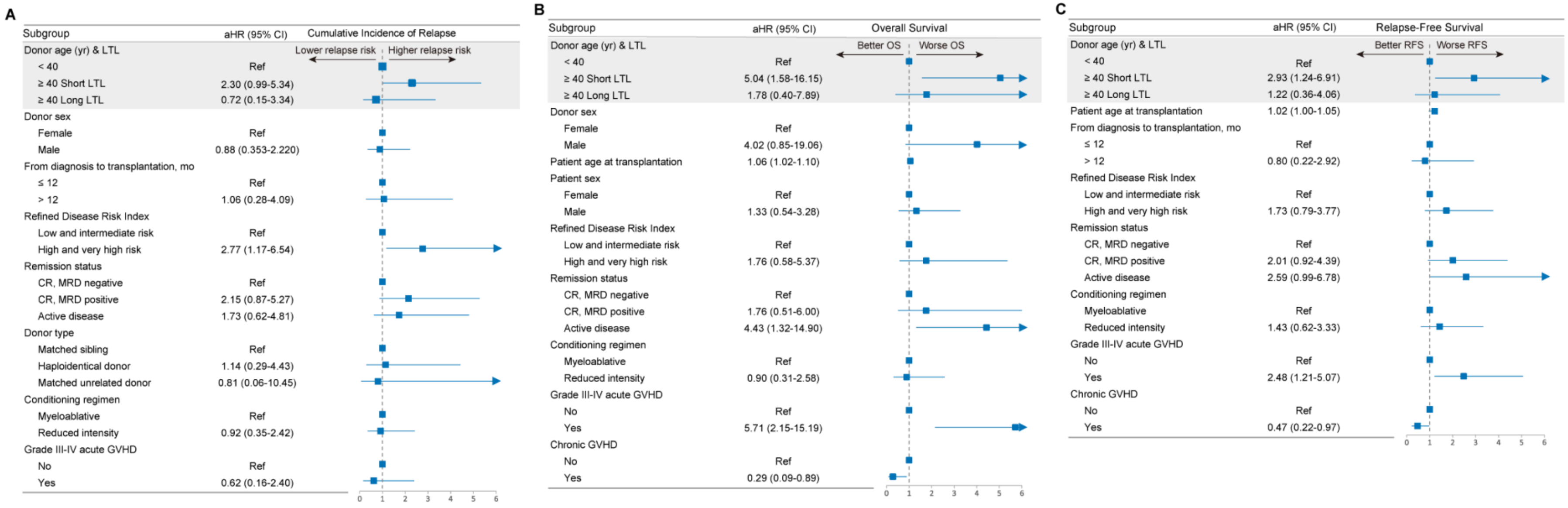

Supplement: Document S2. Figures S1–S6 [file mmc2.pdf]
