# Supplementary material for: Donor leukocyte telomere length emerges as a prognostic factor for transplantation outcomes
Source: Innovation (Camb). 2025 Oct 24;7(3):101147. doi: 10.1016/j.xinn.2025.101147 (PMC12957556; doi:10.1016/j.xinn.2025.101147)
Supplement: Document S3. Article plus supplemental information [file mmc3.pdf]

ARTICLE Clinical Medicine

OPEN ACCESS

www.the-innovation.org

Donor leukocyte telomere length emerges as a prognostic factor for transplantation outcomes

Hengwei Wu,[1,2,3,11] Jing Yu,[1,2,3,11] Yang Cao,[4,11] Ruowen Wei,[5,11] Wei Shi,[5] Yunxian Yu,[6] Zhuoyue Shi,[1,2,3] Jimin Shi,[1,2,3] Yi Luo,[1,2,3] Jian Yu,[1,2,3] Xiaoyu Lai,[1,2,3] Lizhen Liu,[1,2,3] Yamin Tan,[7] Huarui Fu,[1,2,3] Pengxu Qian,[1,2,3] Wenming Shi,[8] Zhongzheng Zheng,[9,*] Xiaoxia Hu,[10,*] He Huang,[1,2,3,*] and Yanmin Zhao[1,2,3,*]

*Correspondence: zzz@catb.org.cn (Z.Z.); hu_xiaoxia@126.com (X.H.); huanghe@zju.edu.cn (H.H.); yanminzhao@zju.edu.cn (Y.Z.)

Received: March 27, 2025; Accepted: October 19, 2025; Published Online: October 24, 2025; https://doi.org/10.1016/j.xinn.2025.101147

© 2025 The Authors. Published by Elsevier Inc. on behalf of Youth Innovation Co., Ltd. This is an open access article under the CC BY license (http://creativecommons.org/licenses/by/4.0/).

GRAPHICAL ABSTRACT

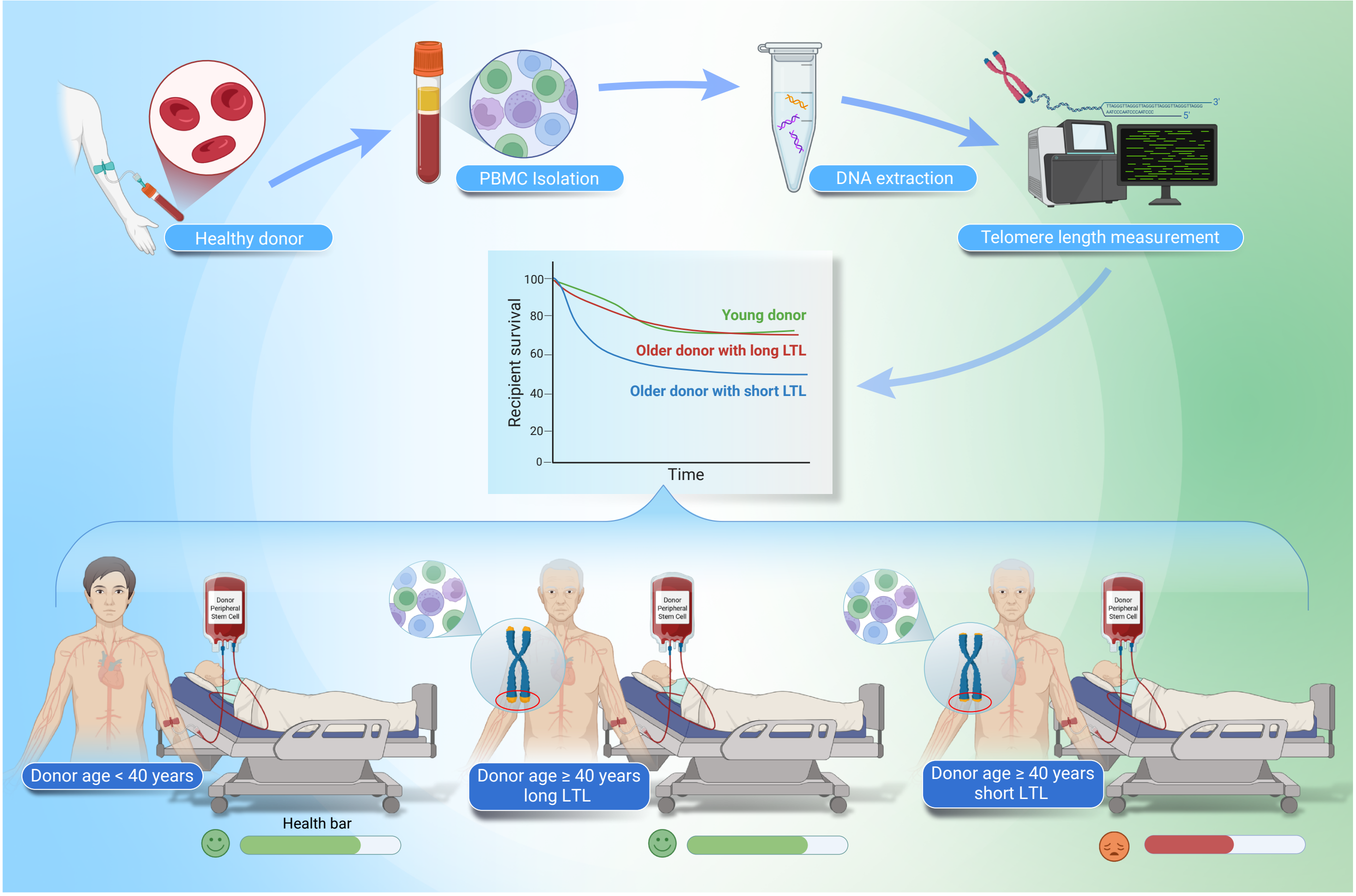

PUBLIC SUMMARY

- Donor leukocyte telomere length relates to recipient outcomes after allogeneic stem cell transplantation.
- Longer donor telomere length associates with lower relapse risk and better overall survival.
- Telomere length may guide donor selection when younger donors (<40 years) are unavailable.

Donor leukocyte telomere length emerges as a prognostic factor for transplantation outcomes

Hengwei Wu,[1,2,3,11] Jing Yu,[1,2,3,11] Yang Cao,[4,11] Ruowen Wei,[5,11] Wei Shi,[5] Yunxian Yu,[6] Zhuoyue Shi,[1,2,3] Jimin Shi,[1,2,3] Yi Luo,[1,2,3] Jian Yu,[1,2,3] Xiaoyu Lai,[1,2,3] Lizhen Liu,[1,2,3] Yamin Tan,[7] Huarui Fu,[1,2,3] Pengxu Qian,[1,2,3] Wenming Shi,[8] Zhongzheng Zheng,[9,*] Xiaoxia Hu,[10,*] He Huang,[1,2,3,*] and Yanmin Zhao[1,2,3,*]

[1]Bone Marrow Transplantation Center of the First Affiliated Hospital & Liangzhu Laboratory, Zhejiang University School of Medicine, Hangzhou 310006, China
[2]Institute of Hematology, Zhejiang University, Hangzhou 310058, China
[3]Zhejiang Province Engineering Research Center for Stem Cell and Immunity Therapy, Hangzhou 311121, China
[4]Department of Hematology, Tongji Hospital, Tongji Medical College, Huazhong University of Science and Technology, Wuhan 430030, China
[5]Institute of Hematology, Union Hospital, Tongji Medical College, Huazhong University of Science and Technology, Wuhan 430022, China
[6]Department of Epidemiology and Statistics, School of Public Health, Zhejiang University, Hangzhou 310058, China
[7]Department of Hematology, Cancer Hospital of the University of Chinese Academy of Sciences, Hangzhou 310022, China
[8]School of Public Health, Li Ka Shing Faculty of Medicine, The University of Hong Kong, Hong Kong 9990077, China
[9]Shanghai Tissuebank Precision Medicine Co., Ltd, Shanghai 201110, China
[10]Shanghai Institute of Hematology, State Key Laboratory of Medical Genomics, National Research Center for Translational Medicine at Shanghai, Ruijin Hospital, Shanghai Jiao Tong University School of Medicine, Shanghai 200025, China
[11]These authors contributed equally
*Correspondence: zzz@catb.org.cn (Z.Z.); hu_xiaoxia@126.com (X.H.); huanghe@zju.edu.cn (H.H.); yanminzhao@zju.edu.cn (Y.Z.)
Received: March 27, 2025; Accepted: October 19, 2025; Published Online: October 24, 2025; https://doi.org/10.1016/j.xinn.2025.101147
© 2025 The Authors. Published by Elsevier Inc. on behalf of Youth Innovation Co., Ltd. This is an open access article under the CC BY license (http://creativecommons.org/licenses/by/4.0/).
Citation: Wu H., Yu J., Cao Y., et al., (2026). Donor leukocyte telomere length emerges as a prognostic factor for transplantation outcomes. The Innovation **7(3)**, 101147.

Younger donors are preferred in allogeneic hematopoietic stem cell transplantation (allo-HSCT), but this preference may limit access for patients whose only available donors are older. This study evaluated whether donor leukocyte telomere length (LTL), in conjunction with donor age, refines donor selection strategies to improve outcomes in recipients with acute leukemia. The discovery analysis included 1,049 acute leukemia patients who underwent allo-HSCT at the First Affiliated Hospital of Zhejiang University School of Medicine, while the replication cohort included 153 recipients from three additional centers. Donor LTL was measured using real-time quantitative PCR and categorized into quartiles: short (Q1) and long (Q2-Q4). Donor age was prespecified as <40 (younger) vs. ≥40 (older) years based on clinical practice. Long donor LTL was associated with a lower cumulative incidence of relapse (CIR), improved overall survival (OS), and better relapse-free survival (RFS) in allo-HSCT recipients. Recipients of grafts from donors aged ≥40 years with short LTL had higher CIR (adjusted hazard ratio [aHR] = 1.51; 95% confidence interval [CI], 1.04–2.19), worse OS (aHR = 1.76; 95% CI, 1.20–2.58), and worse RFS (aHR = 1.53; 95% CI, 1.06–2.20) compared with recipients of grafts from donors <40 years. Immune cell profiling showed that older donors with short LTL had more $CD8^+CD57^+HLA\text{-}DR^-$ cells. In the replication cohort, recipients of grafts from older donors with long LTL had outcomes comparable with those of recipients with younger donors, while those with older donors and short LTL had inferior outcomes. These findings support considering donor LTL as a supplemental factor during donor evaluation, especially when younger donors are not available.

INTRODUCTION

Telomeres, the protective caps at the ends of chromosomes, are essential for maintaining genomic integrity and stability. These repetitive DNA sequences shorten with each cell division due to the end-replication problem. In human leukocytes, telomere attrition occurs over time, with rates influenced by a range of factors such as age, sex, ancestry, and environmental exposures.[1,2] Critically short telomeres can result in cellular dysfunction, including impaired replication, apoptosis, and senescence, all of which contribute to aging and mortality.[3]

Allogeneic hematopoietic stem cell transplantation (allo-HSCT) is a well-established curative treatment for acute leukemia. In China, the number of allo-HSCTs has increased rapidly, with more than 150 centers performing over 5,000 transplantations annually, primarily for malignant diseases.[4] Studies have found that increasing donor age is associated with poorer outcomes in transplant recipients, and current clinical practice prioritizes younger donors, typically under the age of 40 years,[5,6] underscoring the pivotal importance of age consideration in the donor selection protocol. Recently, attention has shifted to donor leukocyte telomere length (LTL) as an indicator of transplant outcomes.[7] Studies have shown that, in matched unrelated donor transplantation for severe aplastic anemia, longer donor LTL is associated with improved survival rates and reduced infection-related mortality in allo-HSCT recipients.[8,9] However, there is currently limited guidance on incorporating LTL into allo-HSCT donor selection criteria, especially for malignant hematologic diseases such as acute leukemia, highlighting the need for further investigation.

This study aims to clarify the impact of donor LTL on transplant outcomes in patients with acute leukemia undergoing allo-HSCT and to develop a more practical donor selection strategy that incorporates LTL, particularly in the context of real-world donor limitations.

MATERIALS AND METHODS

Study populations and procedures

We established a discovery cohort using data from our center, including 1,701 patients who underwent allo-HSCT at the First Affiliated Hospital of Zhejiang University School of Medicine between January 2015 and June 2022. Additionally, we constructed a replication cohort comprising 245 participants who received allo-HSCT at the following hospitals between January 2020 and June 2022: Ruijin Hospital, affiliated with Shanghai Jiao Tong University School of Medicine, Tongji Hospital, and Union Hospital, both affiliated with Tongji Medical College, Huazhong University of Science and Technology. The strategy to construct the research cohort is illustrated in Figure S1. The transplantation protocols are described in detail in Table S1.

Eligibility criteria included: (1) age ≥5 years, (2) first allo-HSCT from matched related, unrelated, or haploidentical donors, (3) achievement of complete remission after transplantation, and (4) availability of donor blood samples before granulocyte colony-stimulating factor mobilization. Exclusion criteria were: (1) diagnoses other than acute leukemia, (2) failure to achieve full donor chimerism following allo-HSCT, and (3) unavailability of donor DNA for LTL analysis. All participants provided written informed consent in accordance with the Declaration of Helsinki. Ethical approval was obtained from the ethics review committees of each participating center.

Telomere length measurement

Real-time quantitative PCR was used to evaluate mean LTLs in kilobases (kb) using the Absolute Human Telomere Length Quantification qPCR Assay Kit (ScienCell Research Laboratories, Carlsbad, CA), following the manufacturer's instructions. Mean LTLs were determined using a well-established quantitative PCR method based on the telomere repeat number (T) to a single-copy gene (S) ratio, which was then converted into absolute telomere length in kb using the standard curve provided by the kit. The single-copy gene primer targeted a 100 base pair region on chromosome 17 for data normalization.[10,11]

DNA was extracted from 5 mL of donor peripheral blood mononuclear cells (PBMCs) using the DNeasy Blood & Tissue Kit (QIAGEN, Germany), according to the manufacturer's protocols. PCR reactions included genomic DNA (5 ng/μL), telomere primer, 2× qPCR master mix, and nuclease-free water. Real-time quantitative PCR was performed on the 7500

Real-Time PCR System (Applied Biosystems), with a protocol of 95°C for 10 min, followed by 32 cycles of 95°C for 20 s, 52°C for 20 s, and 72°C for 45 s. All samples were processed in duplicate, with template controls in each run.

Cytometry by time of flight-based immunophenotypes

Cryovials containing PBMC samples were retrieved from liquid nitrogen and thawed in a 37°C water bath for 1 min. The cell suspension was then transferred to a pre-warmed RPMI 1640 medium (Gibco) at 37°C for washing. Cells were collected by centrifugation and washed once with LunaStain cell staining buffer (Polaris Biology, China). This washing step was repeated, and 3×10^6 cells were stained with 10 μL cisplatin reagent (Polaris Biology) for 5 min, followed by a wash with LunaStain staining solution. Fc receptors were blocked with Fc Receptor Blocking Solution (BioLegend) for 10 min at room temperature. Cells were washed again and stained with a heavy metal-labeled membrane antibody mix (Polaris Biology) for 30 min at room temperature. After two washes, cells were stained with Ir-DNA intercalator reagent (Polaris Biology) for 10 min. Stained cells were then resuspended to 5×10^5 cells/mL in LunaAcq acquisition solution (Polaris Biology) for cytometry by time of flight (CyTOF) analysis (Lunarion, Polaris Biology).

CyTOF data were converted to FSC 3.0 file format (Lunarion, Polaris Biology), and manual gating was performed using FlowJo software (BD Biosciences). T-SNE was applied for an overview of the immune compartment, and FlowSOM clustering and metaclustering were used to identify distinct cell subtypes.

Antibody panel: CD127-139La, CD4-141Pr, CD194/CCR4-142Nd, CD196/CCR6-143Nd, CD15-144Nd, IgD-145Nd, CD27-146Nd, CD45RO-147Sm, CD57-148Nd, CD28-149Sm, HLA-DR-151Eu, CD8a-152Sm, CD45RA-153Eu, CD123/IL-3R-154Sm, PD1-155Gd, CD183/CXCR3-156Gd, CD11c-158Gd, CD16-159Tb, CD19-160Gd, CD24-161Dy, CD185/CXCR5-162Dy, CD25/IL-2RA-163Dy, CD14-164Dy, TCRγδ-165Ho, CCR7-167Er, CD38-170Er, CD45-171Yb, CD66b-174Yb, and CD3-175Lu.

Study endpoints

The primary endpoints were the cumulative incidence of relapse (CIR), overall survival (OS), and relapse-free survival (RFS). CIR was defined as the time from transplantation to the reappearance of leukemia in patients previously in complete remission, with censoring at the last confirmed relapse-free status. The definition of complete remission is provided in the supplemental information (Document S1). OS was defined as the time from transplantation to death from any cause, censored at the last known follow-up for survival. RFS was defined as the time from transplantation to either relapse or death, whichever occurred first, with censoring at the last known follow-up free from relapse or death.

Additional endpoints included engraftment rate, graft-vs.-host disease (GVHD), non-relapse mortality (NRM), and GVHD-free, relapse-free survival (GRFS). Steroid-naive acute GVHD was evaluated and graded according to the consensus guidelines of the Mount Sinai Acute GVHD International Consortium, with a censoring date of 100 days post-transplant. Chronic GVHD was evaluated and graded using the National Institutes of Health criteria, with censoring at the last known follow-up free from chronic GVHD. NRM was defined as death from causes other than relapse, with censoring at the last known follow-up free from relapse. GRFS was defined as the time from transplantation to the occurrence of grade III–IV acute GVHD, chronic GVHD requiring systemic therapy, relapse, or death, with censoring at the last known follow-up without any such events.

Statistical analysis

Data analysis was conducted from May to June 2024. Descriptive statistics were reported as medians with interquartile ranges (IQR) or percentages. Univariate analyses employed the Pearson χ^2 test, Fisher's exact test, Student's t test, or Kruskal-Wallis test as appropriate. To account for multiple testing, p values were adjusted using the Benjamini-Hochberg method.

Survival functions were evaluated using the Kaplan-Meier method, with differences analyzed via the log rank test. The cumulative incidence estimator was used for engraftment, NRM, CIR, and GVHD, with Gray's test applied for CIR and NRM as competing events. Risk factors for CIR were determined using the Fine-Gray proportional hazards model. Cox proportional hazards regression models were used for univariable and multivariable analyses of OS, RFS, and GRFS. In the multivariable analysis, variables significant at $p < 0.1$ in univariable analyses, along with clinically relevant variables[12] (including the positive measurable residual disease before transplant, patient body mass index, the presence of acute and chronic GVHD), and LTL categories (short and long), were included in the Cox or Fine-Gray model to obtain adjusted hazard ratios (aHRs) with 95% confidence intervals (CIs). Proportional hazards assumptions were assessed for all time-to-event models.

Prespecified subgroup analyses were performed. Subgroup-specific aHRs and 95% CIs comparing long vs. short donor LTL were summarized in forest plots. Effect modification was evaluated by adding multiplicative interaction terms (LTL × each prespecified subgroup variable, e.g., donor age) in a single multivariable model; in the absence of a significant interaction, subgroup differences were not interpreted as effect modification.

The sensitivity analysis used the restricted cubic spline function for multivariable analysis to evaluate the association between donor LTL/age and recipients' mortality risk. Analyses were performed using IBM SPSS Statistics software version 22.0.01 (IBM, NY) and R statistical software version 3.4.3. A two-sided $p < 0.05$ was considered statistically significant.

RESULTS

Donor and recipient characteristics in the discovery cohort

In the discovery cohort, 1,701 donor-recipient pairs were screened at the First Affiliated Hospital of Zhejiang University School of Medicine, resulting in 1,049 eligible pairs for analysis (Figure 1A). The cohort included 382 female donors (36%) and 667 male donors (64%), with a median age of 34 years (IQR, 25–45). Among the patients, 489 were female (47%) and 560 were male (53%), with a median age of 35 years (IQR, 26–48). Diagnoses included acute myeloid leukemia (54%), acute lymphoblastic leukemia (44%), and mixed phenotype acute leukemia (2%). The median follow-up duration was 2.0 years (IQR, 1.3–3.4). Due to the non-linear association between donor LTL and mortality risk (Figure S2A), and consistent with prior studies,[8,13,14] we grouped donor LTL by quartiles. Baseline characteristics across four donor LTL quartiles (Q1-Q4) are presented in Table S2. Based on preliminary analysis showing that recipients of grafts from Q2-Q4 donors had similar survival outcomes, whereas those receiving grafts from Q1 donors had worse survival (Figure S2B), we grouped donor LTL into short (Q1) and long (Q2-Q4) categories. Detailed baseline patient and donor characteristics by donor LTL (Q1 vs. Q2-Q4) are presented in Table 1. Donors with a short LTL had a higher proportion of males (72% vs. 61%; p = 0.001). The distribution of donor types was similar between the long and short LTL groups. There was a trend for older patients to receive grafts from donors in the long LTL group (median age: 33 vs. 36 years; p = 0.05). Additionally, recipients in the long LTL category received fewer mononuclear cells than those in the short LTL category (median cell number: 12.5×10^8/kg vs. 11.8×10^8/kg; p = 0.003).

Transplantation outcomes in the discovery cohort

To examine the association between donor LTL and transplantation outcomes, we analyzed patient endpoints according to donor LTL categories. Patients receiving grafts from donors with short LTL had significantly higher CIR, and worse OS and RFS compared with those from long LTL donors (Figures 2A–2C). Engraftment rates, GVHD incidence, and NRM were comparable between groups. The long LTL group exhibited a trend toward higher GRFS, although this difference did not reach statistical significance (Figure S3).

In multivariable analyses adjusting for age, sex, disease risk index, donor type, and conditioning regimen, donors with long LTL remained independently associated with lower CIR (aHR = 0.74; 95% CI, 0.55–0.98), improved OS (aHR = 0.71; 95% CI, 0.54–0.93), and better RFS (aHR = 0.75; 95% CI, 0.59–0.97) in recipients (Table 2).

Subgroup analysis of the impact of donor LTL on recipients' CIR, OS, and RFS

We next examined whether the beneficial effect of long donor LTL on recipient outcomes varied across patient subgroups. Interaction tests between donor LTL categories and subgroup variables were not statistically significant (all interaction *p values* > 0.05). Within the ≥40-year age subgroup, long vs. short donor LTL was associated with lower CIR (aHR = 0.55; 95% CI, 0.36–0.86), better OS (aHR = 0.55; 95% CI, 0.37–0.82), and improved RFS (aHR = 0.60; 95% CI, 0.41–0.87) in allo-HSCT recipients, whereas within the <40-year age subgroup these associations were not statistically significant (Figure 3).

Given the prevailing clinical preference for younger donors (<40 years) and the inflection point observed around donor age 40 years in the discovery cohort's restricted cubic spline model (Figure 1B),[5,6] we prespecified 40 years as a clinically and biologically plausible cutoff for subgroup analyses. Among donors aged ≥40 years, associations of long vs. short donor LTL with recipients' CIR, OS, and RFS were statistically significant. Accordingly, we present results for the ≥40-year subgroup alongside the overall estimates and comparisons with the clinically preferred younger donor group.

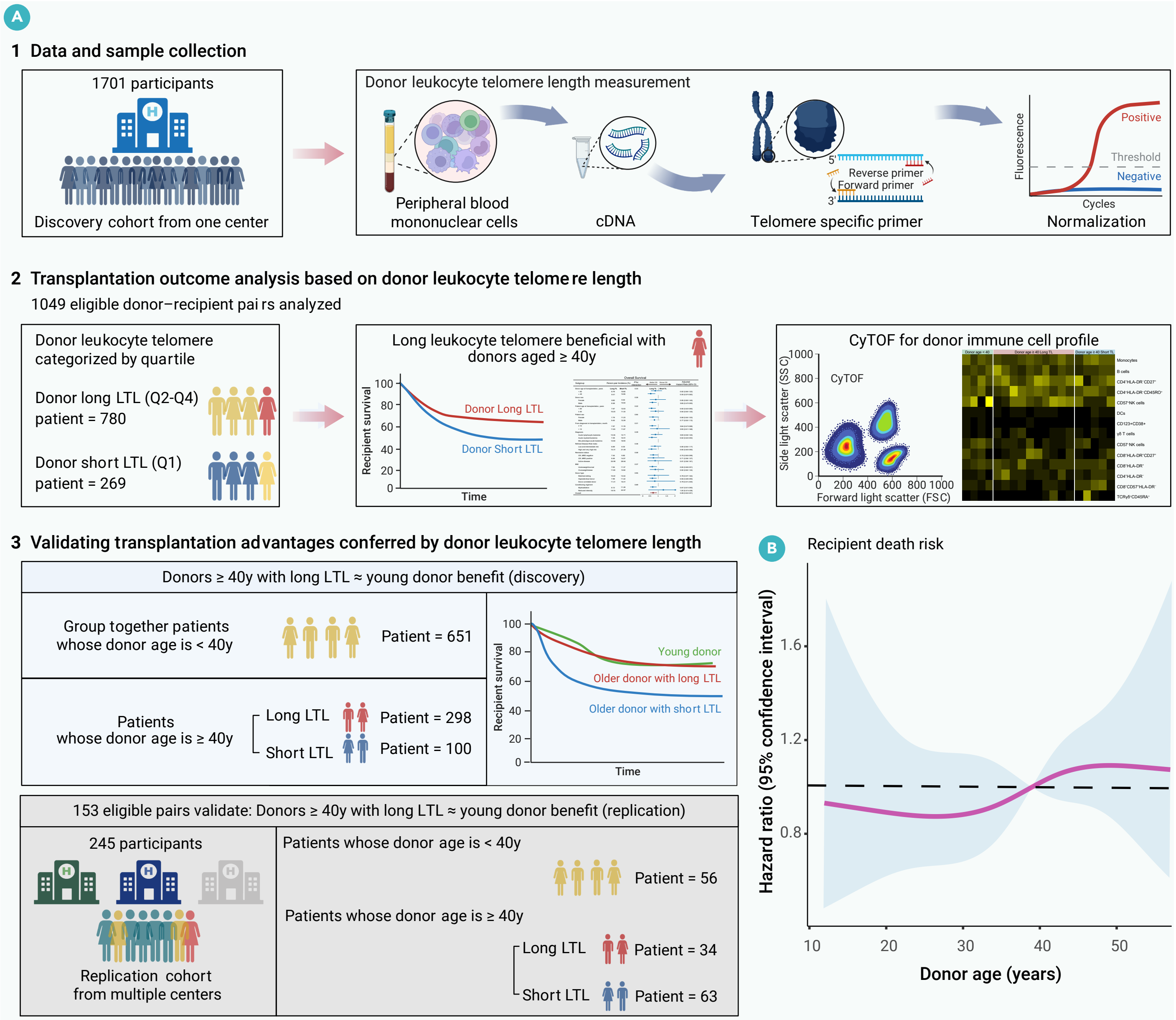

Figure 1. Study profile and donor age association with recipient mortality (A) Overview of patient inclusion in the study, detailing the formation of the discovery and replication cohorts of patients who underwent allogeneic hematopoietic stem cell transplantation for acute leukemia. (B) Restricted cubic spline depicting the association between donor age and recipient mortality risk.

Recipients of grafts from older donors with short LTL experienced significantly worse outcomes than those with younger donors (CIR: 36.9% vs. 26.0%, p = 0.05; OS: 57.2% vs. 71.1%, p = 0.018; RFS: 56.2% vs. 68.3%, p = 0.05). In contrast, recipients of grafts from older donors with long LTL had outcomes comparable to those with younger donors (Figures 4A–4C).

After multivariable adjustment, recipients of grafts from older donors with short LTL had a higher risk of relapse (aHR = 1.51; 95% CI, 1.04–2.19), worse OS (aHR = 1.76; 95% CI, 1.20–2.58), and inferior RFS (aHR = 1.53; 95% CI, 1.06–2.20) compared with recipients with younger donors (Figure 5).

Given the within-subgroup findings in donors ≥40 years, we explored whether telomere-related mutations in older donors contributed to the presence of longer telomeres. However, no specific genetic mutations were identified (Figure S4).

Comprehensive immuno-cell profiling

To explore immunologic correlates of the worse outcomes observed in recipients of grafts from older donors with short LTL, we used CyTOF, a high-dimensional single-cell mass cytometry technique, to profile peripheral blood immune cells from three donor groups: young donors, older donors with long LTL, and older donors with short LTL.

The analysis revealed that the majority of cells in all groups were $CD4^{+}HLA\text{-}DR^{-}CD27^{+}$ cells (13.6% of $CD45^{+}$ cells), followed by $CD57^{+}$ NK cells (12.9% of $CD45^{+}$ cells) (Figures S5A and S5B). T-SNE analysis of $CD45^{+}$ cells showed distinct distributions across groups, with a slight reduction in the $CD8^{+}HLA\text{-}DR^{+}CD27^{+}$ population in older donors with short LTL (Figures S5C and S5D).

Further analysis demonstrated that $CD8^{+}CD57^{+}HLA\text{-}DR^{-}$ T cells were significantly higher in older donors with short LTL compared with those with long LTL (p = 0.034), and marginally higher than in young donors (p = 0.095) (Figure S5E). CD57, a marker of cellular senescence, is associated with age-related immune dysfunction and chronic antigenic stimulation, while HLA-DR indicates cell activation. These observations suggest that older donors with short LTL have a higher frequency of $CD8^{+}CD57^{+}HLA\text{-}DR^{-}$ T cells, a phenotype associated with cellular senescence and reduced activation.

Association of donor LTL with prognosis in the replication cohort

In the replication cohort, 245 donor-recipient pairs from Shanghai Ruijin Hospital, Wuhan Tongji Hospital, and Wuhan Union Hospital were screened,

Table 1. Characteristics of patients and donors by donor leukocyte telomere length (Q1 vs. Q2-Q4) in the discovery cohort

	Short LTL (Q1)	Long LTL (Q2, 3, 4)	*p* value
N	269 (26%)	780 (74%)	–
Donor age at transplantation, years	40 (29–47)	33 (24–43)	<0.001
Donor sex			
Female	74 (28%)	308 (39%)	0.001
Male	195 (72%)	472 (61%)	
Patient age at transplantation, years	33 (24–46)	36 (27–48)	0.05
Patient age at transplantation, years			
<18	30 (11%)	57 (7%)	0.05
≥18	239 (89%)	723 (93%)	
Patient sex			
Female	123 (46%)	366 (47%)	0.78
Male	146 (54%)	414 (53%)	
From diagnosis to transplantation, months	7 (5–10)	7 (5–10)	0.85
Diagnosis			
Acute myeloid leukemia	138 (51%)	427 (55%)	0.29
Acute lymphocytic leukemia	124 (46%)	342 (44%)	
Mixed phenotype acute leukemia	7 (3%)	11 (1%)	
Refined disease risk index			
Low and intermediate risk	187 (70%)	538 (69%)	0.88
High and very high risk	82 (30%)	242 (31%)	
Remission status			
CR, MRD negative	200 (74%)	626 (80%)	0.12
CR, MRD positive	43 (16%)	99 (13%)	
Active disease	26 (10%)	55 (7%)	
Patient BMI at transplantation			
Underweight/normal	187 (70%)	525 (67%)	0.55
Overweight/obese	82 (30%)	255 (33%)	
Donor type			
Matched sibling	35 (13%)	112 (14%)	0.46
Haploidentical donor	212 (79%)	587 (75%)	
Matched unrelated donor	22 (8%)	81 (10%)	
Conditioning regimen			
Myeloablative	250 (93%)	723 (93%)	>0.99
Reduced intensity	19 (7%)	57 (7%)	
Donor-recipient sex			
Female to female	38 (14%)	143 (18%)	0.003
Female to male	36 (13%)	165 (21%)	
Male to male	110 (41%)	249 (32%)	
Male to female	85 (32%)	223 (29%)	
ABO blood type match			

(*Continued on next page*)

Table 1. *Continued*

	Short LTL (Q1)	Long LTL (Q2, 3, 4)	*p* value
Matched	143 (53%)	395 (51%)	0.74
Minor mismatched	54 (20%)	177 (23%)	
Major mismatched	52 (19%)	158 (20%)	
Bidirectional mismatch	20 (7%)	50 (6%)	
Mononuclear cells, $\times 10^8$/kg	12.5 (9.5–18.3)	11.8 (7.7–16.5)	0.003
$CD34^+$ cells, $\times 10^6$/kg	5.2 (3.5–7.3)	5.1 (3.3–6.9)	0.15
Follow-up, years	2.1 (1.3–3.7)	2.0 (1.3–3.3)	0.07
Relapse	71 (26%)	144 (19%)	0.007
Death	81 (30%)	159 (20%)	0.001

Data are *n* (%) or median (IQR [interquartile range]).
Refined disease risk index, CR, and BMI are detailed in the supplemental information.
BMI, body mass index; CR, complete remission; LTL, leukocyte telomere length; MRD, measurable residual disease.

resulting in 153 eligible pairs for analysis (Figure 1A). This cohort included 44 female donors (29%) and 109 male donors (71%), with a median age of 43 years (IQR, 33–50). Among the recipients, 64 were female (42%) and 89 were male (58%), with a median age of 34 years (IQR, 23–47). Diagnoses included acute myeloid leukemia (61%), acute lymphoblastic leukemia (34%), and mixed phenotype acute leukemia (5%). The median follow-up was 2.3 years (IQR, 1.3–2.6). Compared with the discovery cohort, donors in the replication cohort were older (median age: 34 vs. 43 years; $p < 0.001$). Additionally, recipients in the replication cohort received significantly higher doses of mononuclear cells (12.0×10^8/kg vs. 14.0×10^8/kg; $p < 0.001$) and $CD34^+$ cells (5.0×10^6/kg vs. 7.0×10^6/kg; $p < 0.001$) (Table S3).

Donors in the replication cohort were categorized into a younger donor group (age <40 years) and an older donor group (age ≥40 years), with older donors further classified into short and long LTL subgroups based on LTL quartiles from the discovery cohort. Results were consistent with the discovery cohort: recipients of grafts from older donors with long LTL had outcomes similar to those with younger donors, whereas recipients of grafts from older donors with short LTL had higher CIR and poorer OS and RFS (Figures 4D–4F).

The multivariable analysis of the replication cohort, using recipients of grafts from younger donors as the reference group, showed that recipients of grafts from older donors with short LTL had a higher CIR, with the difference approaching statistical significance ($p = 0.051$; Figure S6A), and significantly poorer OS and RFS (Figures S6B and S6C).

DISCUSSION

Recent advances show that telomeres shape cellular aging, disease trajectories, and treatment response. Critically short telomeres activate DNA damage responses that drive senescence or apoptosis, thereby contributing to age-related disease and reduced regenerative capacity.[15,16]

Individuals with longer telomeres in one tissue are likely to have longer telomeres across other tissues as well.[17] Therefore, donors with long LTL tend to also have long telomeres in their hematopoietic stem cells. It is well established that the use of older donors is associated with a higher risk of GVHD and reduced OS, whether in HLA identical or haploidentical transplantation. In hematopoietic stem cells, LTL decreases with age,[18] which suggests that younger donors typically have longer telomeres.[19]

Short LTL in T cells reflects limited replicative potential and is associated with impaired proliferation and enhanced cellular senescence.[20,21] This suggests that shorter LTL may indicate a compromised ability of T cells to perform vital functions such as immune defense and surveillance. Although LTL in this study was measured in PBMCs rather than T cells, PBMCs typically contain a high proportion of T cells (60%–70%).[22,23] In our samples, T cells constituted a mean of

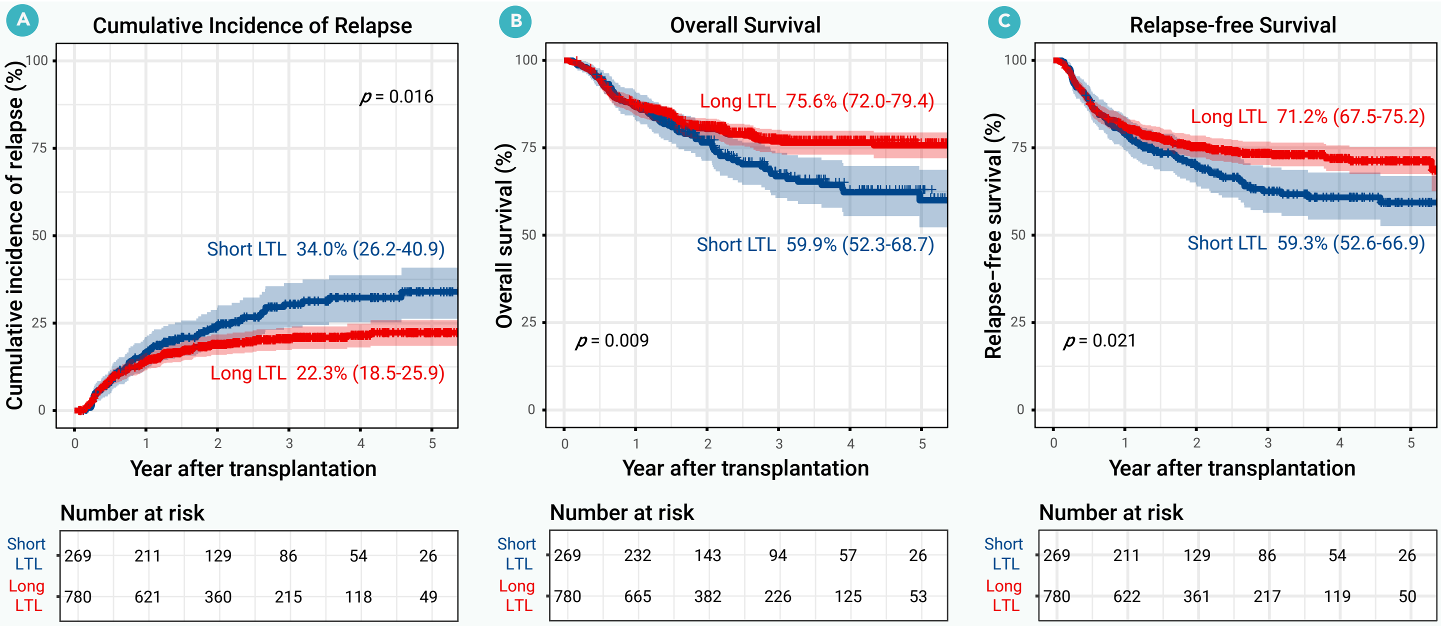

Figure 2. Primary endpoints by donor leukocyte telomere length (discovery cohort) (A) Cumulative incidence of relapse. (B) Overall survival. (C) Relapse-free survival. LTL, leukocyte telomere length.

Table 2. Multivariable analysis results for primary endpoints in the discovery cohort

	Cumulative incidence of relapse	Overall survival	Relapse-free survival
Variable	adjusted hazard ratio (95% CI), *p* value	adjusted hazard ratio (95% CI), *p* value	adjusted hazard ratio (95% CI), *p* value
Donor LTL			
Short LTL	Ref	Ref	Ref
Long LTL	0.74 (0.55–0.98), 0.037	0.71 (0.54–0.93), 0.012	0.75 (0.59–0.97), 0.027
Patient age at transplantation, years	–	1.01 (0.99–1.02), 0.37	1.00 (0.99–1.01), 0.56
From diagnosis to transplantation, months			
≤12	Ref	Ref	Ref
>12	1.20 (0.88–1.65), 0.25	1.04 (0.76–1.42), 0.81	1.14 (0.86–1.51), 0.36
Refined disease risk index			
Low and intermediate risk	Ref	Ref	Ref
High and very high risk	2.17 (1.60–2.94), <0.001	1.91 (1.42–2.56), <0.001	1.85 (1.42–2.42), <0.001
Remission status			
CR, MRD negative	Ref	Ref	Ref
CR, MRD positive	1.74 (1.22–2.47), 0.002	1.45 (1.03–2.04), 0.032	1.51 (1.10–2.07), 0.010
Active disease	2.04 (1.37–3.11), 0.001	1.95 (1.30–2.94), 0.001	1.94 (1.33–2.82), 0.001
Patient BMI at transplantation			
Underweight/normal	/	Ref	Ref
Overweight/obese	–	1.22 (0.93–1.60), 0.15	1.24 (0.97–1.60), 0.09
Donor type			
Matched sibling	Ref	Ref	Ref
Haploidentical donor	0.69 (0.49–0.99), 0.044	0.83 (0.58–1.19), 0.31	0.75 (0.54–1.03), 0.08
Matched unrelated donor	0.71 (0.41–1.20), 0.20	0.98 (0.60–1.60), 0.94	0.82 (0.52–1.30), 0.39
Conditioning regimen			
Myeloablative	Ref	Ref	Ref
Reduced intensity	1.51 (0.95–2.41), 0.08	1.54 (0.97–2.46), 0.07	1.56 (1.02–2.39), 0.042
Grade III-IV acute GVHD			
No	/	Ref	Ref
Yes	–	2.47 (1.67–3.64), <0.001	2.00 (1.39–2.87), <0.001
Chronic GVHD			
No	Ref	Ref	Ref
Yes	0.48 (0.35–0.66), <0.001	0.33 (0.24–0.46), <0.001	0.44 (0.33–0.58), <0.001

Patient age was modeled as a continuous variable.
BMI, body mass index; CI, confidence intervals; CR, complete remission; GVHD, graft-vs.-host disease; LTL, leukocyte telomere length; MRD, measurable residual disease.

56.1% of $CD45^+$ cells. Accordingly, the measured LTL likely reflects T cell telomere length.

Older short LTL donors had a significantly higher proportion of $CD8^+CD57^+HLA\text{-}DR^-$ T cells. CD57 identifies T cell subsets with shortened telomeres (∼2 kb shorter than $CD57^-$ counterparts) and reduced proliferative potential. These senescent-like cells are common in chronic infections and aging, and have impaired effector function.[24,25] In the context of HSCT, such populations might compromise graft-vs.-leukemia activity and are consistent with features of T cell exhaustion. The increased presence of $CD8^+CD57^+HLA\text{-}DR^-$ cells in older short LTL donors, characterized by the absence of HLA-DR (a marker of recent activation), is consistent with an accumulation of "post-effector" clones with reduced anti-leukemic activation. These correlative observations provide a plausible immunologic context for the higher relapse rates observed in recipients of short LTL older donors.

Previous studies in severe aplastic anemia have shown that longer donor LTL correlates with improved recipient OS.[8] A recent study by Gadalla et al. reported a similar association in early-stage leukemia or myelodysplastic syndrome.[26] Consistent with this literature, we observed that longer donor LTL was associated not only with better OS but also with a lower CIR and improved RFS. Given that post-transplant relapse often portends poor prognosis and limited therapeutic options, optimizing donor evaluation to mitigate relapse risk remains an important goal.

In donor age subgroup analyses (<40 vs. ≥40 years), associations of long vs. short donor LTL with lower CIR and better OS/RFS reached statistical significance in donors ≥40 years but did not in donors <40 years.

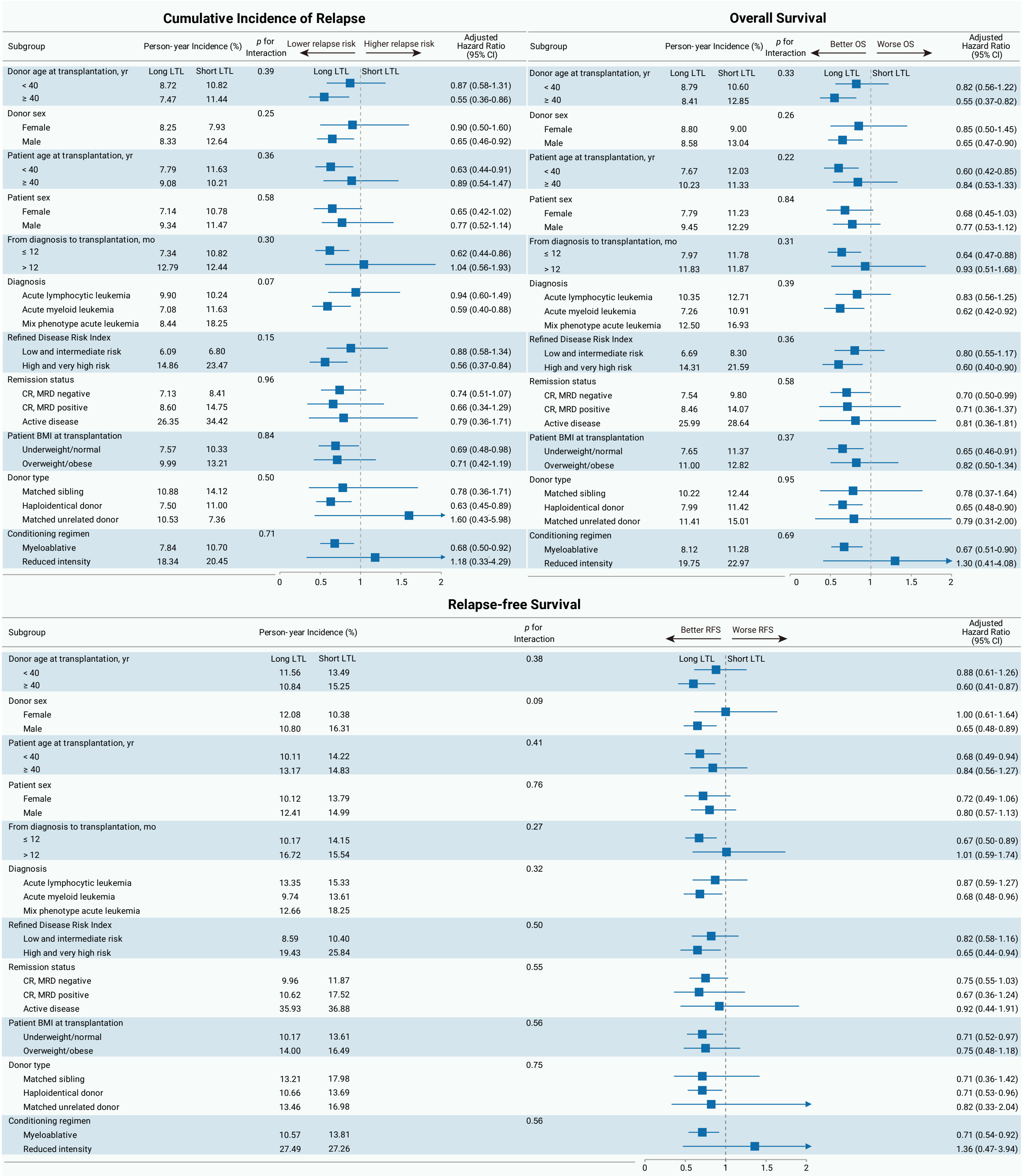

Figure 3. Subgroup analysis forest plot Forest plots show adjusted hazard ratios and 95% CIs for the cumulative incidence of relapse, overall survival, and relapse-free survival across prespecified clinical subgroups. BMI, body mass index; CI, confidence interval; CR, complete remission; MRD, measurable residual disease; LTL, leukocyte telomere length.

When a younger donor is unavailable, measuring donor LTL may provide additional context for donor evaluation. In evaluations that include donors ≥40 years, LTL measurement may help contextualize risk and identify older donors whose expected outcomes resemble those associated with younger donors. Quantitative PCR-based LTL testing is low-cost, requires minimal sample input, and can be completed promptly, making it feasible to incorporate into routine donor evaluations without delaying transplantation.

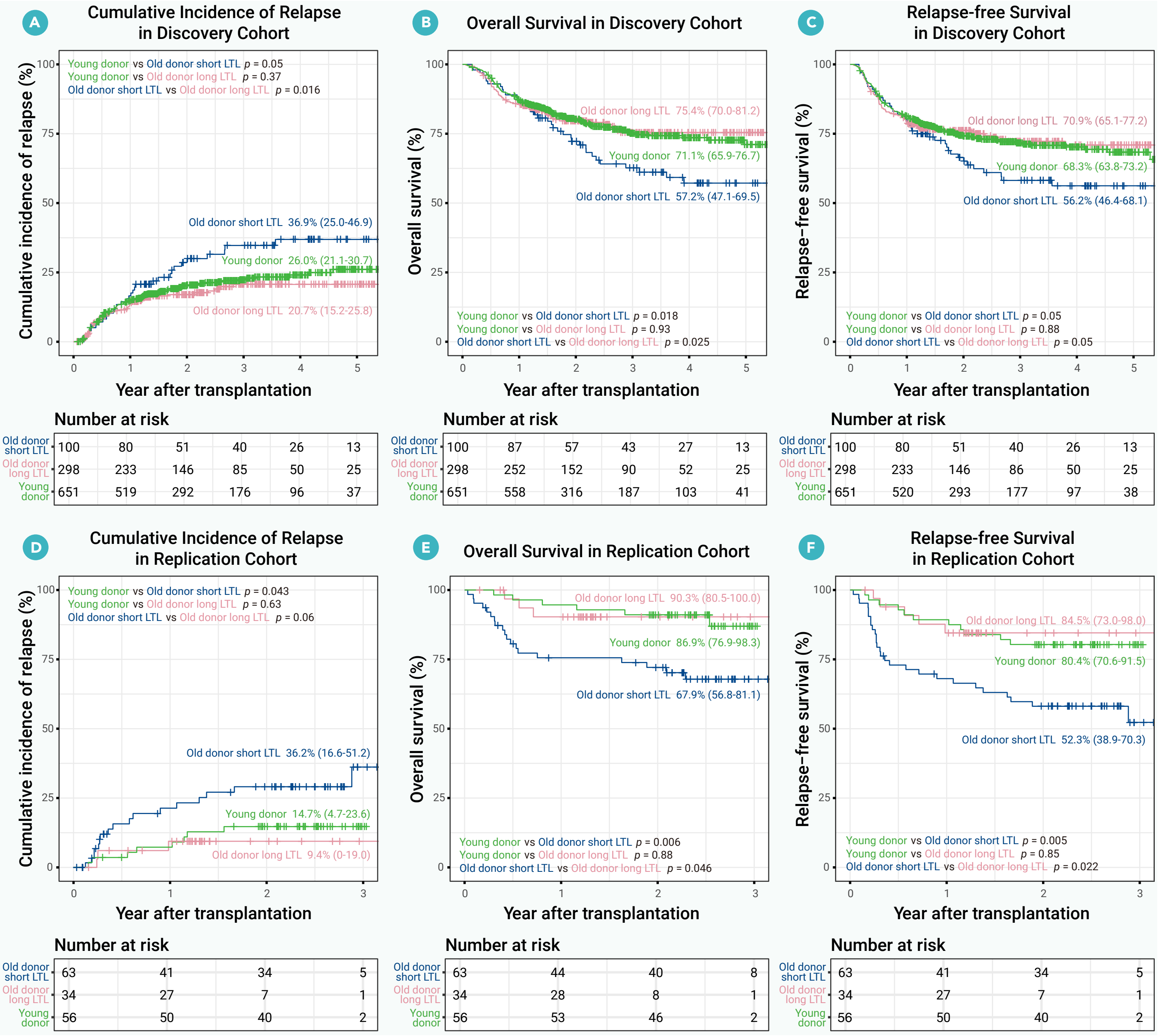

Figure 4. Primary outcomes in recipients grouped by donor age and LTL (A–F) Primary endpoints across three donor groups: younger donors (<40 years), older donors (≥40 years) with long LTL (LTL > 6.92 kb), and older donors (≥40 years) with short LTL (LTL ≤ 6.92 kb). (A–C) Show the cumulative incidence of relapse, overall survival, and relapse-free survival in the discovery cohort. (D–F) Present the corresponding outcomes in the replication cohort. LTL, leukocyte telomere length.

Several limitations should be acknowledged. First, LTL vary among immune cell subtypes.[9,27] Since our measurements were based on donor PBMCs, which include a heterogeneous mix of T, B, and NK cells, further studies are needed to examine telomere length in specific subpopulations. This may help clarify the biological underpinnings of the observed associations with longer LTL. Second, this study was conducted at centers in China, where allo-HSCT practices (particularly the frequent use of haploidentical donors) differ from other settings, which may limit the generalizability of our findings to settings where matched donors are standard. In addition, we did not measure recipient LTL, which may also influence immune recovery and transplant outcomes. The interplay between donor and recipient telomere profiles remains poorly understood and warrants further investigation. Moreover, in related donor settings, shared genetic or environmental factors could influence donor LTL, potentially confounding its association with post-transplant outcomes. Due to the retrospective nature of this study and the limited availability of genomic and lifestyle data, we were unable to comprehensively evaluate these factors. Future studies incorporating paired donor-recipient telomere profiling and integrated genomic and exposomic data will be essential to clarify these complex interactions.

Our study found that donor LTL plays a significant role in transplantation outcomes for acute leukemia patients. Interestingly, we found that older donors with long LTL can have transplantation results similar to younger donors. Measuring donor LTL may provide an adjunctive context for donor evaluation. This is particularly relevant when a younger donor is unavailable.

RESOURCE AVAILABILITY

Materials availability

This study did not generate new unique materials/reagents.

Data and code availability

- The data can be made available upon reasonable request by contacting the corresponding author via email.
- This study does not generate the original code.

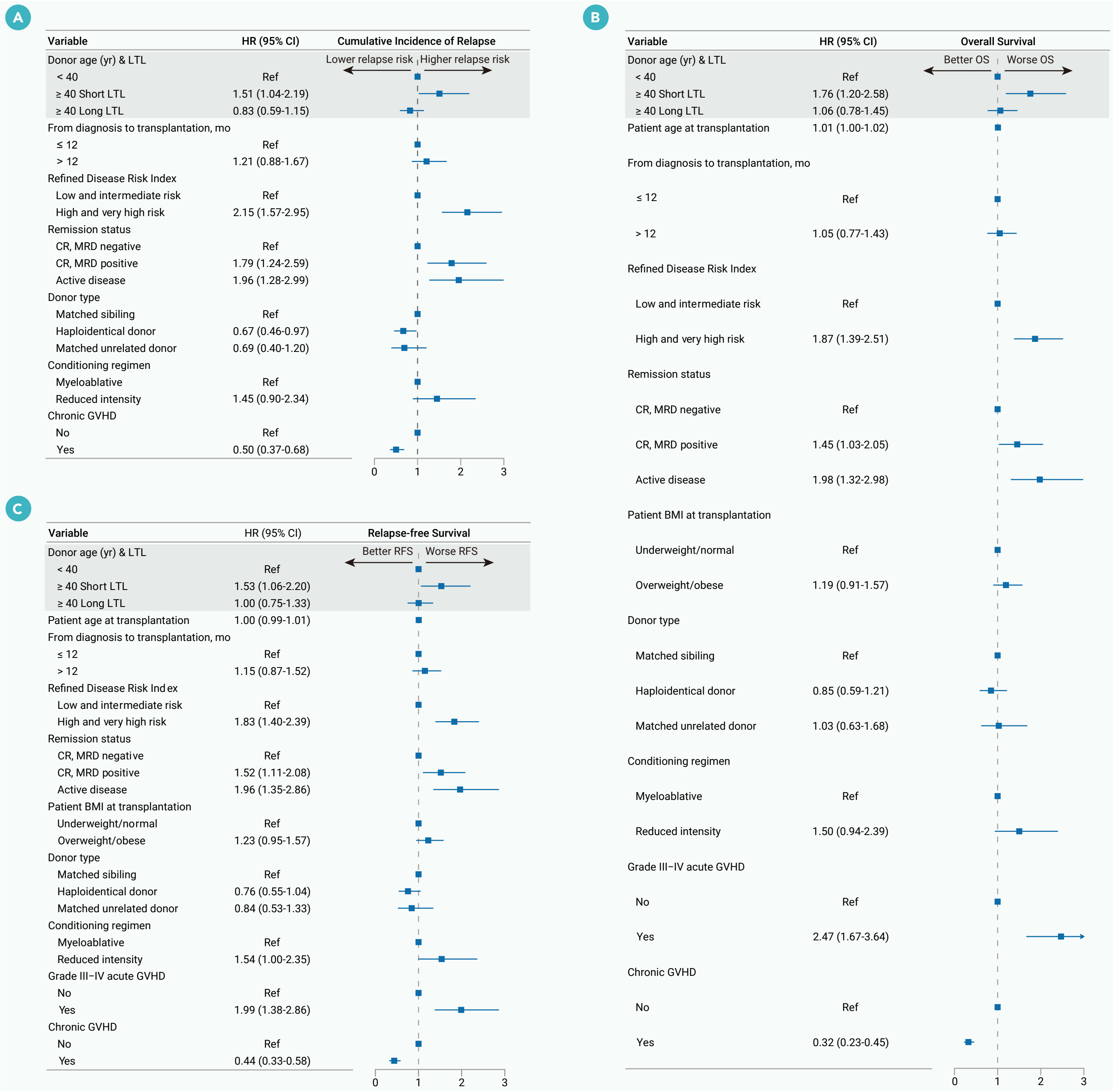

Figure 5. Multivariable models for the primary endpoints of recipient (discovery cohort) (A–C) (A) Fine-Gray proportional hazard model for analyzing the cumulative incidence of relapse. Multivariable Cox regression models for (B) overall survival and (C) relapse-free survival. BMI, body mass index; CI, confidence interval; CR, complete remission; HR, hazard ratio; GVHD, graft-vs.-host disease; LTL, leukocyte telomere length; MRD, measurable residual disease.

FUNDING AND ACKNOWLEDGMENTS

This study was funded by the National Key Research and Development Program of China (2022YFA1103500) and the Natural Science Foundation of Zhejiang Province (LQN25H080004). The funders had no role in study design, data collection and analysis, decision to publish, or preparation of the manuscript. We are grateful to all participants in this trial, including patients, their families, caregivers, and the study team and investigators at all the study sites. Special thanks to Polaris Biology for their technical support in CyTOF analysis.

AUTHOR CONTRIBUTIONS

Y.Z., H.H., X.H., and Z.Z. contributed to the conception and design of the study. H.W., Y.Z., Y.C., R.W., Wei Shi, J.S., Y.L., Jian Yu, X.L., L.L., Y.T., H.F., and X.H. provided study materials or patients. H.W., Y.Z., Y.C., and R.W. contributed to the manuscript writing. H.W. and Jing Yu contributed to the data analysis and interpretation. Y.Y. and Wenming Shi provided administrative support for statistical analyses. All authors contributed to the manuscript and approved the final version.

DECLARATION OF INTERESTS

Z.Z., as an employee, has received research funding from Shanghai Tissuebank Precision Medicine Co., Ltd.

SUPPLEMENTAL INFORMATION

It can be found online at https://doi.org/10.1016/j.xinn.2025.101147.

REFERENCES

1. Ye, Q., Apsley, A.T., Etzel, L. et al. (2023). Telomere length and chronological age across the human lifespan: A systematic review and meta-analysis of 414 study samples including 743,019 individuals. *Ageing Res. Rev.* **90**:102031. DOI:10.1016/j.arr.2023.102031
2. Demanelis, K., Jasmine, F., Chen, L.S. et al. (2020). Determinants of telomere length across human tissues. *Science (New York, N.Y.)* **369**:eaaz6876. DOI:10.1126/science.aaz6876
3. Armanios, M. and Blackburn, E.H. (2012). The telomere syndromes. *Nat. Rev. Genet.* **13**:693–704. DOI:10.1038/nrg3246
4. Xu, L.P., Lu, D.P., Wu, D.P. et al. (2023). Hematopoietic Stem Cell Transplantation Activity in China 2020-2021 During the SARS-CoV-2 Pandemic: A Report From the Chinese Blood and Marrow Transplantation Registry Group. *Transplant. Cell. Ther.* **29**:136.e1–136.e7. DOI:10.1016/j.jtct.2022.11.011
5. Canaani, J., Savani, B.N., Labopin, M. et al. (2018). Donor age determines outcome in acute leukemia patients over 40 undergoing haploidentical hematopoietic cell transplantation. *Am. J. Hematol.* **93**:246–253. DOI:10.1002/ajh.24963
6. DeZern, A.E., Franklin, C., Tsai, H.-L. et al. (2021). Relationship of donor age and relationship to outcomes of haploidentical transplantation with posttransplant cyclophosphamide. *Blood Adv.* **5**:1360–1368. DOI:10.1182/bloodadvances.2020003922
7. Zhang, X.H., Chen, J., Han, M.Z. et al. (2021). The consensus from The Chinese Society of Hematology on indications, conditioning regimens and donor selection for allogeneic hematopoietic stem cell transplantation: 2021 update. *J. Hematol. Oncol.* **14**:145. DOI:10.1186/s13045-021-01159-2
8. Gadalla, S.M., Wang, T., Haagenson, M. et al. (2015). Association between donor leukocyte telomere length and survival after unrelated allogeneic hematopoietic cell transplantation for severe aplastic anemia. *JAMA* **313**:594–602. DOI:10.1001/jama.2015.7
9. Gadalla, S.M., Aubert, G., Wang, T. et al. (2018). Donor telomere length and causes of death after unrelated hematopoietic cell transplantation in patients with marrow failure. *Blood* **131**:2393–2398. DOI:10.1182/blood-2017-10-812735
10. Canela, A., Vera, E., Klatt, P. et al. (2007). High-throughput telomere length quantification by FISH and its application to human population studies. *Proc. Natl. Acad. Sci. USA* **104**:5300–5305. DOI:10.1073/pnas.0609367104
11. Harley, C.B., Futcher, A.B. and Greider, C.W. (1990). Telomeres shorten during ageing of human fibroblasts. *Nature* **345**:458–460. DOI:10.1038/345458a0
12. Wu, H., Shi, J., Luo, Y. et al. (2022). Assessment of Patient-Specific Human Leukocyte Antigen Genomic Loss at Relapse After Antithymocyte Globulin-Based T-Cell-Replete Haploidentical Hematopoietic Stem Cell Transplant. *JAMA Netw. Open* **5**:e226114. DOI:10.1001/jamanetworkopen.2022.6114
13. Myllymäki, M., Redd, R., Reilly, C.R. et al. (2020). Short telomere length predicts nonrelapse mortality after stem cell transplantation for myelodysplastic syndrome. *Blood* **136**:3070–3081. DOI:10.1182/blood.2020005397
14. Peffault de Latour, R., Calado, R.T., Busson, M. et al. (2012). Age-adjusted recipient pretransplantation telomere length and treatment-related mortality after hematopoietic stem cell transplantation. *Blood* **120**:3353–3359. DOI:10.1182/blood-2012-01-403337
15. Blackburn, E.H. (2005). Telomeres and telomerase: their mechanisms of action and the effects of altering their functions. *FEBS Lett.* **579**:859–862. DOI:10.1016/j.febslet.2004.11.036
16. Sahin, E. and Depinho, R.A. (2010). Linking functional decline of telomeres, mitochondria and stem cells during ageing. *Nature* **464**:520–528. DOI:10.1038/nature08982
17. Friedrich, U., Griese, E., Schwab, M. et al. (2000). Telomere length in different tissues of elderly patients. *Mech. Ageing Dev.* **119**:89–99. DOI:10.1016/s0047-6374(00)00173-1
18. Vaziri, H., Dragowska, W., Allsopp, R.C. et al. (1994). Evidence for a mitotic clock in human hematopoietic stem cells: loss of telomeric DNA with age. *Proc. Natl. Acad. Sci. USA* **91**:9857–9860. DOI:10.1073/pnas.91.21.9857
19. Wagner, W., Bork, S., Horn, P. et al. (2009). Aging and replicative senescence have related effects on human stem and progenitor cells. *PLoS One* **4**:e5846. DOI:10.1371/journal.pone.0005846
20. Najarro, K., Nguyen, H., Chen, G. et al. (2015). Telomere Length as an Indicator of the Robustness of B- and T-Cell Response to Influenza in Older Adults. *J. Infect. Dis.* **212**:1261–1269. DOI:10.1093/infdis/jiv202
21. Lanna, A., Vaz, B., D'Ambra, C. et al. (2022). An intercellular transfer of telomeres rescues T cells from senescence and promotes long-term immunological memory. *Nat. Cell Biol.* **24**:1461–1474. DOI:10.1038/s41556-022-00991-z
22. Autissier, P., Soulas, C., Burdo, T.H. et al. (2010). Evaluation of a 12-color flow cytometry panel to study lymphocyte, monocyte, and dendritic cell subsets in humans. *Cytometry A* **77**:410–419. DOI:10.1002/cyto.a.20859
23. Verhoeckx, K., Cotter, P., López-Expósito, I. et al. (2015). The Impact of Food Bioactives on Health: in vitro and ex vivo Models, Chapter 15 (Springer), pp. 161–167. DOI:10.1007/978-3-319-16104-4_15
24. Cura Daball, P., Ventura Ferreira, M.S., Ammann, S. et al. (2018). CD57 identifies T cells with functional senescence before terminal differentiation and relative telomere shortening in patients with activated PI3 kinase delta syndrome. *Immunol. Cell Biol.* **96**:1060–1071. DOI:10.1111/imcb.12169
25. Brenchley, J.M., Karandikar, N.J., Betts, M.R. et al. (2003). Expression of CD57 defines replicative senescence and antigen-induced apoptotic death of CD8+ T cells. *Blood* **101**:2711–2720. DOI:10.1182/blood-2002-07-2103
26. Gadalla, S.M., Katki, H.A., Lai, T.P. et al. (2025). Donor telomeres and their magnitude of shortening post-allogeneic haematopoietic cell transplant impact survival for patients with early-stage leukaemia or myelodysplastic syndrome. *EBioMedicine* **114**:105641. DOI:10.1016/j.ebiom.2025.105641
27. Bellon, M. and Nicot, C. (2017). Telomere Dynamics in Immune Senescence and Exhaustion Triggered by Chronic Viral Infection. *Viruses* **9**:289. DOI:10.3390/v9100289

The Innovation, Volume 7

Supplemental Information

Donor leukocyte telomere length emerges as a prognostic factor for transplantation outcomes

Hengwei Wu, Jing Yu, Yang Cao, Ruowen Wei, Wei Shi, Yunxian Yu, Zhuoyue Shi, Jimin Shi, Yi Luo, Jian Yu, Xiaoyu Lai, Lizhen Liu, Yamin Tan, Huarui Fu, Pengxu Qian, Wenming Shi, Zhongzheng Zheng, Xiaoxia Hu, He Huang, and Yanmin Zhao

Document S1

Definitions

CR, complete remission

CR is defined as the presence of fewer than 5% leukemic blasts in the bone marrow, with no evidence of leukemic cells in the peripheral blood or at extramedullary sites.

R-DRI, refined Disease Risk Index

R-DRI is a clinically validated stratification system used to predict prognosis in patients undergoing allogeneic hematopoietic stem cell transplantation, particularly those with hematologic malignancies. R-DRI categorizes patients into four risk groups—low, intermediate, high, and very high—based on disease type, disease status, and prognostic factors such as cytogenetic and molecular abnormalities.[S1]

BMI, body mass index

BMI was calculated as weight in kilograms divided by the square of height in meters (kg/m^2). Weight and height were measured by trained healthcare professionals following standardized protocols established by the World Health Organization. Participants were measured without shoes and in light clothing. Weight was recorded to the nearest 0.1 kg using a calibrated beam scale, and height to the nearest 0.2 cm using a portable stadiometer. Each measurement was taken three times, and the average value was used in the calculation. Based on BMI, participants were classified as underweight (< 18.5 kg/m^2), normal weight (18.5–23.9 kg/m^2), overweight (24.0–27.9 kg/m^2), or obese (≥ 28.0 kg/m^2).[S2, S3]

Reference

[S1]. Armand P, Kim HT, Logan BR, et al. Validation and refinement of the Disease Risk Index for allogeneic stem cell transplantation. *Blood*. 2014 Jun 5;123(23):3664-71.

[S2]. WHO Expert Consultation. Appropriate body-mass index for Asian populations and its implications for policy and intervention strategies. *Lancet*. 2004;363:157–163.

[S3]. Zhou BF; Cooperative Meta-Analysis Group of the Working Group on Obesity in China. Predictive values of body mass index and waist circumference for risk factors of certain related diseases in Chinese adults--study on optimal cut-off points of body mass index and waist circumference in Chinese adults. *Biomed Environ Sci*. 2002 Mar;15(1):83-96.

Supplemental Tables

Table S1. Transplantation plan

**Transplantation plan**	
Conditioning regimen	
Myeloablative regimen	ARAC2Bu3Cy2 / Bu4Cy2 / Flu5Bu4 / Flu5Bu2Mel100
Reduced intensity regimen	Flu6Bu3 / Flu6Bu2 / Flu6Mel140–120
T cell depletion regimen (in vivo)	
Haploidentical HSCT	Anti–T lymphocyte globulin (Grafalon®, 10–15 mg/kg, intravenous infusion, days –5 to –2) or anti-thymocyte globulin (Thymoglobulin®, 6–7.5 mg/kg, intravenous infusion, days –5 to –2)
Matched related HSCT	Thymoglobulin® (4.5 mg/kg, intravenous infusion, days –5 to –2) for patients or donors aged > 40 years
Unrelated HSCT	Thymoglobulin® (4.5–7.5 mg/kg, intravenous infusion, days –5 or –4 to –2)
Peripheral blood stem cell mobilization regimen	
All donors	Granulocyte colony-stimulating factor, 7.5–10 μg/kg/day, subcutaneous injection for 4–5 consecutive days, followed by peripheral blood stem cell collection

ARAC, Cytarabine; Bu, Busulfan; Cy, Cyclophosphamide; Flu, Fludarabine; HSCT, hematopoietic stem cell transplantation; Mel, Melphalan.

Table S2. Baseline patient and transplant characteristics by donor leukocyte telomere length quartiles (Q1–Q4) in the discovery cohort

Donor LTL, quartiles	Q1	Q2	Q3	Q4	*p* value
n	269 (26%)	258 (25%)	262 (25%)	260 (25%)	
Donor age at transplantation, yr	40 (29–47)	35 (25–45)	34(25–44)	30 (23–41)	< 0.001
Donor sex					0.002
Female	74 (28%)	91 (35%)	97 (37%)	120 (46%)	
Male	195 (72%)	167 (65%)	165 (63%)	140 (54%)	
Patient age at transplantation, yr	33 (24–46)	34 (25–46)	35 (26–50)	38 (29–49)	0.018
Patient age at transplantation, yr					0.09
< 18	30 (11%)	20 (8%)	25 (10%)	12 (5%)	
≥ 18	239 (89%)	238 (92%)	237 (90%)	248 (95%)	
Patient sex					0.80
Female	123 (46%)	115 (45%)	127 (48%)	124 (48%)	
Male	146 (54%)	143 (55%)	135 (52%)	136 (52%)	
From diagnosis to transplantation, mo	7 (5–10)	7 (5–10)	7 (5–10)	6 (5–10)	0.016
Diagnosis					0.15
Acute myeloid leukemia	138 (51%)	131(51%)	137 (52%)	159 (61%)	
Acute lymphocytic leukemia	124 (46%)	123 (48%)	119 (45%)	100 (39%)	
Mixed phenotype acute leukemia	7 (3%)	4 (2%)	6 (2%)	1 (0%)	
Refined Disease Risk Index					0.09
Low and intermediate risk	187 (70%)	187 (72%)	189 (72%)	162 (62%)	
High and very high risk	82 (30%)	71 (28%)	73 (28%)	98 (38%)	
Remission status					0.013
CR, MRD negative	200 (74%)	192 (74%)	213 (81%)	221 (85%)	
CR, MRD positive	43 (16%)	49 (19%)	32 (12%)	18 (7%)	
Active disease	26 (10%)	17 (7%)	17 (7%)	21 (8%)	

Patient BMI at transplantation					0.80
Underweight/normal	187 (70%)	176 (68%)	170 (65%)	179 (69%)	
Overweight/obese	82 (30%)	82 (32%)	92 (35%)	81 (31%)	
Donor type					0.80
Matched sibling	35 (13%)	34 (13%)	36 (14%)	42 (16%)	
Haploidentical donor	212 (79%)	195 (76%)	201 (77%)	191 (74%)	
Matched unrelated donor	22 (8%)	29 (11%)	25 (10%)	27 (10%)	
Conditioning regimen					0.75
Myeloablative	250 (93%)	242 (94%)	246 (94%)	235 (90%)	
Reduced intensity	19 (7%)	16 (6%)	16 (6%)	25 (10%)	
Donor-recipient sex					0.020
Female to female	38 (14%)	41 (16%)	48 (18%)	54 (21%)	
Female to male	36 (13%)	50 (19%)	49 (19%)	66 (25%)	
Male to male	110 (41%)	93 (36%)	86 (33%)	70 (27%)	
Male to female	85 (32%)	74 (29%)	79 (30%)	70 (27%)	
ABO blood type match					0.76
Matched	143 (53%)	129 (50%)	137 (52%)	129 (50%)	
Minor mismatched	54 (20%)	62 (24%)	56 (21%)	59 (23%)	
Major mismatched	52 (19%)	56 (22%)	52 (20%)	50 (19%)	
Bidirectional mismatch	20 (7%)	11 (4%)	17 (7%)	22 (8%)	
Mononuclear cells, × 10^8/kg	12.5 (9.5–18.3)	12.6 (8.5–17.3)	12.3 (8.5–17.7)	10.1 (6.8–14.7)	< 0.001
$CD34^+$ cells, × 10^6/kg	5.2 (3.5–7.3)	5.3 (3.9–7.6)	5.1 (3.4–6.8)	4.5 (3.1–6.6)	0.018
Follow-up, yr	2.1 (1.3–3.7)	2.3 (1.3–3.6)	2.1 (1.3–3.2)	1.6 (1.2–2.9)	< 0.001
Relapse	71 (26%)	47 (18%)	48 (18%)	49(19%)	0.05
Death	81 (30%)	53 (21%)	55 (21%)	51 (20%)	0.025

NOTE. Data are n (%) or median (IQR[interquartile range]). *p* values were false discovery rate-adjusted (Benjamini–Hochberg correction).

BMI, body mass index; CR, complete remission; LTL, leukocyte telomere length; MRD, measurable residual disease.

Table S3. Patient and donor characteristics in the discovery vs. replication cohort

	Discovery cohort N = 1049	Replication cohort N = 153	*p* value
Donor age at transplantation, yr	34 (25–45)	43 (33–50)	< 0.001
Donor sex			0.06
Female	382 (36%)	44 (29%)	
Male	667 (64%)	109 (71%)	
Patient age at transplantation, yr	35 (26–48)	34 (23–47)	0.16
Patient sex			0.27
Female	489 (47%)	64 (42%)	
Male	560 (53%)	89 (58%)	
From diagnosis to transplantation, mo	7 (5–10)	6 (4–8)	< 0.001
Diagnosis			0.011
Acute myeloid leukemia	565 (54%)	94 (61%)	
Acute lymphocytic leukemia	466 (44%)	52 (34%)	
Mixed phenotype acute leukemia	18 (2%)	7 (5%)	
Refined Disease Risk Index			0.54
Low and intermediate risk	725 (69%)	102 (67%)	
High and very high risk	324 (31%)	51 (33%)	
Remission status			0.010
CR, MRD negative	826 (79%)	104 (68%)	
CR, MRD positive	142 (13%)	29 (19%)	
Active disease	81 (8%)	20 (13%)	
Patient BMI at transplantation			0.77
Underweight/normal	712 (68%)	102 (67%)	
Overweight/obese	337 (32%)	51 (33%)	
Donor type			0.16
Matched sibling	147 (14%)	20 (13%)	
Haploidentical donor	799 (76%)	125 (82%)	
Matched unrelated donor	103 (10%)	8 (5%)	
Conditioning regimen			0.05
Myeloablative	973 (93%)	135 (88%)	
Reduced intensity	76 (7%)	18 (12%)	
Donor-recipient sex			0.047
Female to female	181 (17%)	18 (12%)	
Female to male	201 (19%)	37 (24%)	
Male to male	359 (34%)	63 (41%)	
Male to female	308 (30%)	35 (23%)	
ABO blood type match			0. 55
Matched	538 (51%)	83 (54%)	
Minor mismatched	231 (22%)	26 (17%)	
Major mismatched	210 (20%)	34 (22%)	
Bidirectional mismatch	70 (7%)	10 (7%)	
Mononuclear cells, × 10^8/kg	12.0 (8.0–17.0)	14.0 (11.0–18.1)	< 0.001

CD34⁺ cells, × 10^6/kg	5.0 (3.1–7.0)	7.0 (5.0–9.0)	< 0.001
Follow-up, yr	2.0 (1.3–3.4)	2.3 (1.3–2.6)	0.06
Relapse	215 (21%)	28 (18%)	0.53
Death	240 (23%)	28 (18%)	0.20

Data are n (%) or median (IQR[interquartile range]).
BMI, body mass index; CR, complete remission; MRD, measurable residual disease.
